# A Review on Removal and Destruction of Per- and Polyfluoroalkyl Substances (PFAS) by Novel Membranes

**DOI:** 10.3390/membranes12070662

**Published:** 2022-06-27

**Authors:** Suman Das, Avner Ronen

**Affiliations:** Zuckerberg Institute for Water Research, The Jacob Blaustein Institutes for Desert Research, Ben-Gurion University of the Negev, Sede-Boqer Campus 84990, Israel; sumand@post.bgu.ac.il

**Keywords:** PFAS, nanofiltration, reverse osmosis, novel membranes, hybrid membranes, coupled technology

## Abstract

Per- and Polyfluoroalkyl Substances (PFAS) are anthropogenic chemicals consisting of thousands of individual species. PFAS consists of a fully or partly fluorinated carbon–fluorine bond, which is hard to break and requires a high amount of energy (536 kJ/mole). Resulting from their unique hydrophobic/oleophobic nature and their chemical and mechanical stability, they are highly resistant to thermal, chemical, and biological degradation. PFAS have been used extensively worldwide since the 1940s in various products such as non-stick household items, food-packaging, cosmetics, electronics, and firefighting foams. Exposure to PFAS may lead to health issues such as hormonal imbalances, a compromised immune system, cancer, fertility disorders, and adverse effects on fetal growth and learning ability in children. To date, very few novel membrane approaches have been reported effective in removing and destroying PFAS. Therefore, this article provides a critical review of PFAS treatment and removal approaches by membrane separation systems. We discuss recently reported novel and effective membrane techniques for PFAS separation and include a detailed discussion of parameters affecting PFAS membrane separation and destruction. Moreover, an estimation of cost analysis is also included for each treatment technology. Additionally, since the PFAS treatment technology is still growing, we have incorporated several future directions for efficient PFAS treatment.

## 1. Background on PFAS

Per- and Polyfluoroalkyl Substances (PFAS) are anthropogenic chemicals consisting of thousands of individual species. Resulting from their hydrophobic and oleophobic nature and chemical and mechanical stability, PFAS have been used extensively worldwide since the 1940s as oil- and water-repellent products, mainly in non-stick household items, paints, food packaging, cosmetics, lubricants, electronics, and aviation film-forming foam (AFFF) for firefighting [1]. PFAS consist of a carbon chain with carbon–fluorine (C–F) bonds which require a high amount of energy to break (536 kJ/mole) [2], resulting in stable compounds that are difficult to degrade naturally; therefore, they remain present in the environment for long durations [3].

Exposure to PFAS may result in health issues such as hormonal imbalances, liver disfunction, a compromised immune system, cancer, fertility disorders, negative effects on fetal growth, and learning ability in children [4]. Exposure routes are by inhaling, ingesting, and direct skin contact [5]. Furthermore, ultra-short chain PFAS (C ≤ 2, e.g., C_2_F_6_, CHF_3_, CF_4_, etc.) are volatile as well as highly water-soluble, and can easily enter the human body when breathing or consuming food or drinking water [6,7]. The adverse health effects of PFAS are not only limited to humans; they could be equally harmful to animals and livestock [4].

One of the main exposure routes to PFAS is by wastewater effluents [1] from industries manufacturing them or from municipal wastewater impacted by PFAS-related products. As PFAS are not easily removed by conventional biological wastewater treatment processes such as activated sludge [8], effluents were shown to contain varying PFAS species which could contaminate the aquatic environment. In the US, wastewater effluents were shown to contain ƩPFAS_29_: 20–4773 ng/L (from 29 PFAS species) in North Carolina [9] and ƩPFAS_17_: 442–2234 ng/L in Nevada [10]. Similar data was obtained in Europe, where ƩPFAS_8_: 1.2–290 ng/L was obtained in the Netherlands [11,12], ƩPFAS_8_: 0.3–90.4 ng/L was found in Germany [12], and ƩPFAS_8_: 1.7–200 ng/L was found in Italy [12].

As the awareness of the presence and environmental and health impacts of PFAS has been growing, several countries have published guidelines and regulations addressing PFAS concentrations in drinking water [13]. The US Environmental Protection Agency has set the PFOA and PFOS (individually or combined) limit in drinking water to 70 ng/L [14], whereas for the UK, Germany, Italy, Netherlands, and Sweden, it is 10, 300, 30–500, 200–390, and 90 ng/L, respectively [15].

**Figure 1:**
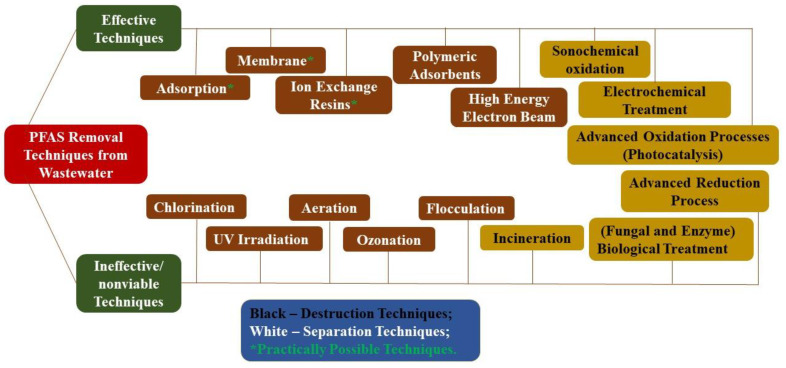
Techniques for removing Per- and Polyfluoroalkyl Substances (PFAS) from wastewater.

Resulting from their stability, PFAS were shown to have limited biodegradation in environmental conditions and low environmental concentrations [16]. Biodegradation was shown only in specific cases of co-metabolism [17,18]. Therefore, the destruction or removal of PFAS is mainly based on high-energy incineration [19] or advanced oxidation processes, e.g., electrochemical oxidation, microwave treatment, photocatalytic degradation [20,21], pyrolysis, plasma-based treatment [22], and sonochemical reactions [23] (Figure 1). As PFAS degradation techniques are energy extensive, they are relatively expensive, especially considering the large volume and high flow rate of wastewater and groundwater requiring treatment. Among the techniques mentioned, membrane-based treatment can concentrate low concentrations of PFAS present in the wastewater/groundwater and is therefore one of the most effective treatment techniques in terms of cost and efficiency [24,25,26].

In recent years, a growing number of studies have addressed the environmental occurrence, fate, and transport of PFAS in potable water and wastewater treatment plants, as well as varying treatment approaches [2,27,28,29,30,31]. Therefore, it is necessary to systematically review and critically analyze the state of knowledge and determine research gaps for suitable and economically viable technology leading to PFAS removal from water. This study thoroughly reviews existing publications to summarize the currently available membrane-based treatments and identifies novel approaches for PFAS removal. It also addresses factors affecting PFAS removal via membrane filtration and delineates research gaps and key future research directions.

## 2. Current Treatment Approaches

Conventional water/wastewater treatment technologies (e.g., flocculation, aeration, sand/rapid filtration, sedimentation, and disinfection) were shown to be less efficient in treating PFAS-contaminated groundwater and wastewater [32]. As PFAS are typically found in the aqueous phase at relatively low concentrations in the range of tens to hundreds of ng/L, they require concentration by adsorption, ion exchange resins, and membrane filtration prior to destruction. The advantages and limitations of the above-mentioned PFAS concentration techniques are summarized in Table 1.

### 2.1. Adsorption and Ion Exchange

Adsorption is considered an effective separation technique for PFAS due to its low cost, high efficiency, simple operation, and insensitivity toward toxic substances. Besides conventional adsorbent materials (SI Table S1)—e.g., granular and powdered activated carbon—few other efficient adsorbents— such as silica [35], zeolites [48], aminated rice husk [49], graphitized carbon nitride—g-C_3_N_4_ [50], metal organic frameworks—MOFs [51,52], covalent organic frameworks—COFs [53], and modified chitosan [54]—have been synthesized and used. PFAS adsorption mainly depends on two predominant forces, namely electrostatic [55] and hydrophobic iterations [56,57,58]. Adsorption efficiency also depends on the molecular structure of PFAS and its physiochemical properties (surface functional group, porosity, polarity, diameter, surface charge, and surface area). Furthermore, the solution’s pH [59] and ionic strength [60] may also impact the adsorption rate and capacity. When used for large-scale applications, adsorption processes have a few limitations, including the relatively high cost of the adsorbent, which is required in high volumes. Moreover, the regeneration of the adsorbent by chemical [61], microwave [62], or thermal treatment [63] is expensive, and the adsorbent may lose its effectiveness after several regeneration cycles. Additionally, short-chain PFAS (<C_6_) are difficult to remove by conventional adsorbers, e.g., based on activated carbon.

Ion exchange resins are also used for PFAS concentration and removal. Recent studies (SI Table S2) show that PFAS removal by ion exchange resins is an efficient technology, especially for short-chain PFAS. PFAS removal efficiency depends on the resin’s functional group (e.g., tertiary, or quaternary amine), polymer matrix, and porosity (e.g., gel, macro porosity). PFAS removal capacity by resins can also be affected by other parameters, such as pH, ionic strength, and the presence of organic matter and inorganic salts [41,64,65,66]. Regenerating and disposing of used resins are among the main downsides of ion exchange resins for PFAS removal. Studies reveal that regenerated resins are less efficient in adsorbing PFAS and require a longer contact time than single-use resins [31,40,67]. Furthermore, the chemical regeneration process of the resins is expensive; therefore, the destruction of resins is a better option to avoid the loss [68,69].

### 2.2. Membrane Separation

In contrast to adsorption by activated carbon or removal by ion exchange resins, the removal capability of membranes is usually not limited by organic matter concentration, salts, or the presence of co-contaminants, as membrane selectivity is defined by its surface properties, such as porosity, pore size, material and zeta potential [70,71,72]. Furthermore, the presence of a ‘fouling’ layer on the membrane may even enhance its selectivity and removal efficiency while reducing permeate flux [73,74]. The membranes can be divided into porous and dense membranes according to their physical characteristics.

In terms of porous membranes, in most cases the relatively large pore size—in the range of tens of nm to microns of porous membranes (e.g., microfiltration and ultrafiltration)—makes them less efficient for PFAS separation [25]. For example, Appleman et al. (2014) [32] observed that the removal of PFOS, PFDoA, and FOSA by a UF/MF membrane system was ineffective (removal of 24%, 44%, and 42%, respectively), resulting from the membrane’s large pore size in comparison to the PFAS compounds (molecular weight 499–614 g/mol), leading to limited size exclusion [32]. Another work on UF membranes (UP020 and UH030) by Zeng et al. (2017) [75] showed 68.9–83.7% rejection of PFHxA for similar reasons. Additional work by Olimattel et al. (2021) [45] shows that modification of a commercially available UF membrane (UA60) via a layer-by-layer approach with polyelectrolytes (polyallylamine hydrochloride and polyacrylic acid) reduced the membrane’s molecular weight cut off (MWCO) from 2263 Da to 1411 Da and the porosity of the membrane by 9.2%. This functionalization increased PFOA and PFOS removal efficiency by 30% compared to the unmodified membrane. In addition, the presence of cations (e.g., Mg^2+^ (1 mM added to 10 ppm humic acid) and Ca^2+^ (2 mM added to 10 ppm humic acid)) and humic acid (5–10 ppm) in the treated water might also impact PFAS separation, resulting in the formation of macromolecular complexes (cation-PFOS, PFOA-cation-humic acid, PFOS-cation-humic acid, etc.). This was shown to increase the removal efficiency of PFOA and PFOS by 18% and 23%, respectively [45], in UF membranes.

**Figure 2:**
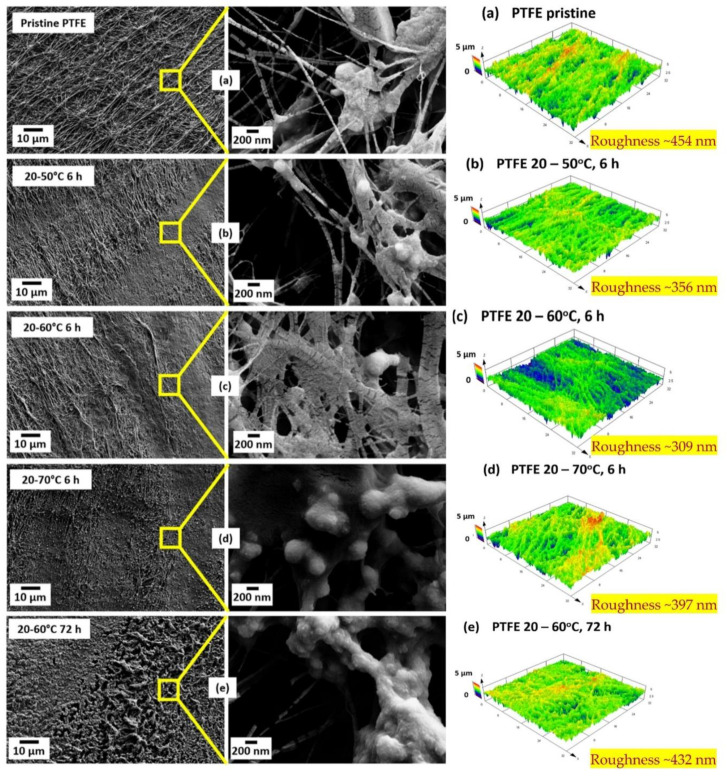
Scanning Electron Microscopy (SEM) and Atomic Force Microscopy (AFM) images of PTFE membrane in Membrane Distillation (MD) of PFPeA (**a**) before filtration, (**b**) after filtration of 6 h at 50 °C, (**c**) 60 °C and (**d**) 70 °C feed temperatures, and (**e**) 60 °C feed temperatures with 72 h duration of distillation process; the permeate temperature was maintained at 20 °C for all the experiments (reprinted with permission from Ref. [24]. Copyright 2020 Elsevier).

**Figure 3:**
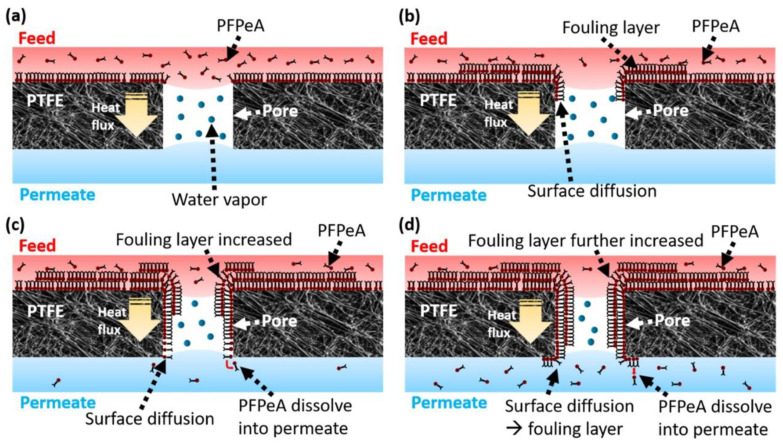
Mechanism of membrane distillation process for PFAS (PFPeA) removal: (**a**) nucleation of surface deposition, (**b**) PFAS adsorption and aggregation across the membrane pores, (**c**) surface diffusion within the membrane pores, and (**d**) PFPeA coating within the pores (reprinted with permission from Ref. [24]. Copyright 2020 Elsevier).

Another approach for PFAS separation using porous membranes is membrane distillation (MD). MD requires that a hydrophobic MF membrane is hydrophobic with a relatively large pore size (0.1–0.45 µm), and the transport of water is based on partial water pressure resulting from a thermal gradient [76]. Chen et al. (2020) [24] used direct contact MD to remove perfluoropentanoic acid (PFPeA). They used a commercially available poly-(tetrafluoroethylene) (PTFE) membrane with an average pore size of 0.46 ± 0.02 μm. Their work mainly shows the impact of membrane surface fouling and material stability, which depends on the amphiphilic nature of the PFPeA (Figure 2). SEM and AFM images in Figure 2 illustrate the membrane’s surface morphology under different conditions. The long-term use of the membrane was affected by the surface diffusion across the membrane, as can be confirmed from the AFM analysis through the change in surface roughness in Figure 2. When feed temperatures were increased from 50 to 70 °C, permeate flux increased from 17 to 43 kg/m^2^/h; on the other hand, rejection was reduced from 85 to 58%, leading to an increase in PFPeA concentration by 1.8, 2.1, and 2.8 times in permeate as feed temperature increases to 50, 60, and 70 °C, respectively. A simple mechanism of PFAS (PFPeA) separation by membrane distillation is depicted in Figure 3. First, (a) at time t = 0, the surface deposition starts taking place upon flowing of the PFAS solution in contact with the membranes. In the next step, (b) adsorption rises and produces globular aggregates across the membrane pores. Following this, (c) surface diffusion occurs within the membrane pores. Finally, (d) the membrane gets saturated and pores blocking or filling take place due to PFPeA deposition within the pores. As PFAS are amphiphilic, they act as surfactants and might impact the hydrophobicity of the MD membrane, leading to a wetting of the pores and thus limited rejection. In addition, as a result of the need to continuously heat the feed, MD suffers from temperature polarization [77] and is considered cost inefficient [24].

In contrast to porous membranes, the use of dense membranes, e.g., in nanofiltration (NF) and reverse osmosis (RO), is a viable, sustainable, and highly efficient technique for removing emerging contaminants from water and wastewater [26,78]. Therefore, commercial/industrial removal of PFAS from groundwater is typically done by RO [79]. Most studies suggest that RO membranes are superior to NF membranes in terms of PFAS removal efficiency and that they can achieve PFAS discharge values established by the USA, Canada, Australia, and European countries. As all membranes, including RO, are prone to fouling, they are a suitable choice for removing PFAS mainly from groundwater considered less contaminated (e.g., low TSS, low TOC) [80]. Tang et al. (2007) [81] compared the efficiency of NF (DK, NF270, and NF90) and RO (BW30, LFC3, ESPA3, SG, and LFC1) membranes in removing PFOS from wastewater; the results reveal that the rejection of RO membrane efficiencies was above 99%, whereas for NF membranes, removal efficiency varied between 90–99%. Other studies also confirm the removal of multiple PFAS species (PFOS, PFHxA, PFOA, PFDA, etc.) by RO membranes [75,82,83], with over 99% rejection compared to the around 95% rejection of NF (NF270, NF200, DK, NTR-7410, NTR-7450, and DL) [75,78,84] membranes.

Rejection of other pollutants such as colloids (foam, gel, muddy water, etc.) and large organic compounds (carbohydrates, lipids, proteins, etc.) by NF membranes is mainly influenced by physical sieving [85,86]. However, for ions (e.g., Ca^2+^, Mg^2+^, Na^+^, HCO_3_^−^, etc.) and lower molecular weight substances (methanol, isopropanol, etc.) [87], solution diffusion and the surface charge (zeta potential) of the membrane and pollutant play a key role in separation. Due to their varying length and charge, the removal of PFAS by NF is described in the literature by multiple mechanisms, including:

Steric (size) exclusion—rejection depends on the molecular weight cut-off (MWCO) of the membrane and dominates throughout the separation process. Lower MWCO leads to higher rejection of PFAS, resulting from the steric hindrance that affects the rejection of short/large molecules of PFAS [88,89]. For example, NF membranes with varying MWCO, e.g., NF270 with 300 Da and NF90 with 100–200 Da, showed rejection of 96.2 and 99.8%, respectively, for PFHxA [75]. Furthermore, the presence of ions (Ca^2+^, Na^+^, Mg^2+^, Fe^3+^) in the feed water leads to the formation of complex compounds containing PFAS. This typically resulted in a higher rejection of PFAS resulting from the larger size of the complex. In addition, during the NF/RO membrane filtration process, fouling of the membrane by organics and colloids may lead to a change in membrane selectively resulting from partial pore blocking, leading to further reduction of pore size, resulting in a higher rejection of PFAS [58]. In contrast, fouling was also shown to lead to foulant-enhanced concentration polarization, resulting in lower rejection of pollutants [90].

Electrostatic interaction: The interaction between the charged organic pollutants and the charged membrane surface resulting from electrostatic forces (Donnan effect) is affected by pH and ionic strength. Filtration of anionic PFAS by negatively charged membranes resulted in higher separation of PFAS, especially for short-chain PFAS [44].

Solution-diffusion: PFAS separation is based on diverse diffusivities and solubility in the NF/RO membrane matrix. In this case, PFAS are ‘dissolved’ in the NF/RO thin-film and diffuse across the membrane down a concentration gradient. The separation of different PFAS species present in the wastewater can be achieved based on the compounds with different diffusivities and solubility in the membrane matrix [91,92].

NF membranes were shown to be able to remove PFAS due to their small pore diameter, low MWCO, and negative surface charge [93,94]. Multiple reported studies on NF claim above 93–95% removal efficiency. At the same time, other impurities are also removed along with PFAS, and the concentrated brine solution must be subjected to further treatment [84,95,96].

Franke et al. (2019) [78] showed the use of nanofiltration (NF270) to reject a mixture of 15 different PFAS species with carbon chains of C_4_–C_12_ (e.g., PFHxS, PFBS, PFOS, PFHxA, etc.), with an initial concentration of 6–110 ng/L (molecular weight 213–500 g/mol). Accordingly, the removal efficiency was 99%, with a feed flow rate of 2.3 m^3^ h^−1^ [78]. An additional NF membrane (NF90) was shown to have >98% removal efficiency for 32 different PFAS species (C_3_–C_8_, PFHxA, PFOA, PFBS, PFOS, PFHxS, etc. Average concentration: ~160 ng/L) [38]. This NF membrane successfully removed other pollutants, such as uranium-238, dissolved organic carbon, and mineral hardness, from the raw water. This work also highlights the treatment cost, which largely depends on the drinking water treatment targets and concentrates discharge requirement [38]. In the above-mentioned NF membranes (NF90 [38] and NF270 [78]) studies, NF90 showed less rejection compared to NF270, even though NF90 has a lower MWCO. This is probably because 32 different species of PFAS (mostly short-chain, C_3_–C_8_) were separated using NF90, whereas NF270 was employed to remove 15 different types of PFAS (mostly longer chain, C_4_–C_12_). Another study by Liu et al. confirms the PFAS’ rejection of >97% by NF, while 42 different PFAS were present in the medium. Their study also revealed that the operating conditions of membranes marginally impacted the rejection of PFAS and that long-term use of a membrane is also possible [97].

Another polyamide thin-film composite membrane (spiral wound NF/RO) [97] for AFFF containing PFAS treatment was employed by Liu et al. (2021). The rejections by these membranes were >97% for most of the operating conditions (Flux: 7–50 LMH, Feed flowrate: 5.7–13.2 Lpm, feed pressure: ~60 psi) and water matrices (laboratory matrix (~60,000 ng/L) and groundwater (~6000 ng/L)). The shorter-chain PFAS in groundwater showed a rejection of 92–95% in some cases; in these, it was affected by Ca^2+^ ions and dissolved organic matters, which may have interacted with the sulfonate groups present in the system and reduced rejection. The organic matters and Ca^2+^ ions present in the ground water can also deposit on the membrane surface (sometimes it forms a complex of organic matter-Ca^2+^) and reduce the membrane surface charge (it becomes less negative), which leads to less electrostatic repulsion between the membrane surface and PFAS; finally, the membrane becomes less efficient. Furthermore, these ions and organic matter successfully decrease the PFAS rejection due to fouling enhanced concentration polarization, where the solute concentration on the membrane surface is greater than in the bulk wastewater. However, the membrane rejection was ~98% even after 13 days of uninterrupted operation, which shows its stability and efficacy for a long-term run [97].

On the other hand, RO membranes were shown to be highly efficient in PFAS removal due to their highly selective polyamide thin film. The rejection of fluorinated compounds by RO membranes can be explained through the solution diffusion model, where PFAS molecules have a very slow diffusion rate through the membrane’s active thin film compared to the water molecules [82,83,98,99,100].

Flores et al. (2013) [82] were able to remove >99% of fluorinated compounds (PFOA and PFOS) present in a wastewater matrix by RO. After the RO process, trace amounts of PFOA (<4.2–5.5 ng/L) and PFOS (3–21 ng/L) were detected in the treated water. These concentrations are lower than the US recommended value in drinking water (70 ng/L). In another work by Tang et al. (2006) [83], semiconductor wastewater with a wide range of PFOS concentrations (0.5–1500 mg/L) was treated by RO membranes (ESPA3, LFC3, BW30, and SG), resulting in over 99% rejection. In addition, Zeng et al. (2017) [75] were also able to remove PFHxA (>99% rejection) from wastewater using RO membranes (NTR-759 HR). Accordingly, most of the PFAS removal processes by RO membranes suggest that the treated effluents can reach PFAS values below the drinking water limitations suggested.

Current examples of NF and RO systems used for PFAS removal are presented in Table 2.

The separation techniques of PFAS can be affected by multiple parameters, hence it is necessary to know those parameters for a better understanding of the process. The most important parameters are discussed in the section below.

## 3. Factors Controlling PFAS Separation by Membranes

PFAS rejection by membrane technology can be impacted through several parameters. To enhance or optimize the process performance, it is necessary to understand the basic parameters that control and affect membrane treatment efficiency. Here, we present some of the crucial parameters.

**Effect of organic matter:** Organic matter present in the feed solution can influence PFAS treatment by coupling or reacting with the targeted PFAS compound, changing the membrane surface charge and leading to membrane fouling. Most of the reported work investigated the effect of fulvic acid and/or humic acid as an organic matter in simulated wastewater containing PFAS. However, the industrial wastewater matrix has a wide range of organic (proteins, amino acids, humic acids, fulvic acid, carboxylic acid, etc.) or inorganic (heavy metals, sulfur, phosphorous, etc.) compounds along with varying PFAS compounds. Organic matter was shown to bind to the membrane’s surface and lead to fouling. In consequence, the permeate flux decreases, and the rejection rate may increase. Moreover, fouling caused by organic matter deposition onto a composite membrane can impact the membrane’s surface charge, turning it more hydrophilic, which may not favor the removal of large chain PFAS.

**Effect of pH**: Changes in the solution’s pH may impact the membrane’s surface charge according to the isoelectric point of the compounds and the relevant groups at the membrane’s surface. Furthermore, in some cases, the membrane’s pore size, flux, and rejection rate can be manipulated by changing the pH [103,104]. Depending on the functional groups at the membrane’s surface and their pKa values, the membrane’s surface may be positively charged, which attracts anionic PFAS (negatively charged), resulting in a decrease in rejection. On the other hand, if the membrane surface is negatively charged, the electrostatic interaction between anionic PFAS and negatively charged membrane can enhance PFAS separation efficiency [28]. In some cases, the membrane’s pore size can be manipulated by controlling the solution’s pH, which affects PFAS removal [105]. The electrostatic repulsion force inside the membrane pores may be reduced when the pH is lowered, which leads to membrane pore size shrinkage and results in enhanced rejection rate [106].

**Effect of ions and ionic strength:** The interaction between different ions (present in water and on the membrane surface) and PFAS is a critical factor. Due to electrostatic interaction between PFAS and ions present in the water, ions can bind PFAS to form larger clusters which may even lead to partial pore-blocking [107]. This is expected to reduce the transport of short and long-chained PFAS through the membrane, resulting in a better PFAS removal. In addition, the increase of valance ions present in the water matrix can improve the electrostatic interaction between PFAS and ions, which promotes the PFAS clustering. For instance, the presence of PO_4_^3−^ resulted in better removal efficiency in comparison with Cl^−^ or SO_4_^2−^ when assessing the removal of positively charged compounds. This phenomenon is not only limited to anions, and the presence of divalent and trivalent cations (Ca^2+^, Pb^2+^, Fe^3+^) can also enhance PFAS rejection.

**Effect of chain length and hydrophobicity:** PFAS are widely used for their hydrophobic and oleophobic nature. Long-chain PFAS are considered more hydrophobic than short-chain PFAS due to the presence of a longer hydrophobic ‘tail’. During membrane filtration of PFAS containing wastewater, PFAS are prone to interacting with solid surfaces (e.g., membranes) present in the system. Therefore, hydrophobic membranes may have an added value when used to remove PFAS by coupling filtration and adsorption [78,91,108].

**Effect of membrane zeta potential:** The zeta potential of a membrane is a function of surface-attached groups (e.g., carboxyl, amine groups) and of the ionic strength. Increasing the ionic strength typically results in a reduction in membrane surface charge as a result of ‘shrinkage’ of the electrostatic double layer and masking of surface charge. As a result, lower membrane surface charge leads to weak electrostatic repulsive forces between similar charged PFAS and membranes and resulting in a reduced rejection rate [109]. Accordingly, maximum rejections by electrostatic forces may be achieved by increasing the charge density at the membrane surface, thus leading to higher electrostatic repulsion forces [91].

**Effect of membrane surface properties:** Among different NF membranes, organic membranes have an advantage over inorganic membranes, resulting from easier processing, appropriate robustness, and low cost [91]. The active layer of the membrane is a key component in quantifying the PFAS removal performance. The NF membrane surface is usually negatively charged and hydrophilic, along with having a low molecular weight cutoff, which affects the membrane performance. During the separation process, PFAS concentrate (micelles or hemi-micelles) can form on the membrane surface due to concentration polarization. PFAS micelles are formed at relatively high concentrations [110]. A critical micelles concentration of PFOA and PFOS ranges between 25–38 mM and 8 mM, respectively [111].

The formation of micelles was shown to reduce the PFAS rejection caused by fouling enhanced concentration polarization (CP), where the solute concentration at the membrane surface is remarkably higher than in the bulk solution [97].

In the case of PFAS removal via an adsorption-based process coupled with membranes, the membrane’s surface is coated with adsorbent materials and the process is controlled by the characteristics of adsorbents, such as adsorbent particle size, surface area, pore-volume, etc. In such cases, the electrostatic force between the membrane surface and the PFAS mostly depends on the adsorbent material and not the membrane’s properties [112].

**The initial concentration of PFAS:** The initial concentration of the PFAS in the feed affects both permeate flux and removal efficiency.

Several reported studies [80,113] addressed the removal efficiency of PFAS when treating a high feed concentration (100 ppm). A high concentration of PFAS in the feed may lead to micelle formation when concentrations reach the critical micelle concentration. While in typical wastewater and groundwater streams, it is very unlikely that the critical micelle concentration will be reached; this should be noted when treating concentrated PFAS streams such as industrial effluents.

Since membrane technology for PFAS removal has several benefits over other available techniques (adsorption, ion exchange resins, etc.), we will review and discuss aspects and modern techniques related to membrane technology in the next section.

## 4. Novel Membranes for PFAS Rejection and Removal

The use of NF and RO membranes can efficiently remove PFAS from water but may be impacted by fouling or require high pressure/energy to achieve appropriate separation [114]. Various modifications have been suggested to improve the membrane’s performance and longevity, including the development of novel membranes and surface modification of commercial membranes, which play a critical role in PFAS rejection [113]. Novel membranes developed for PFAS removal and treatment are described below:

### 4.1. Polymeric Membranes

**Figure 4:**
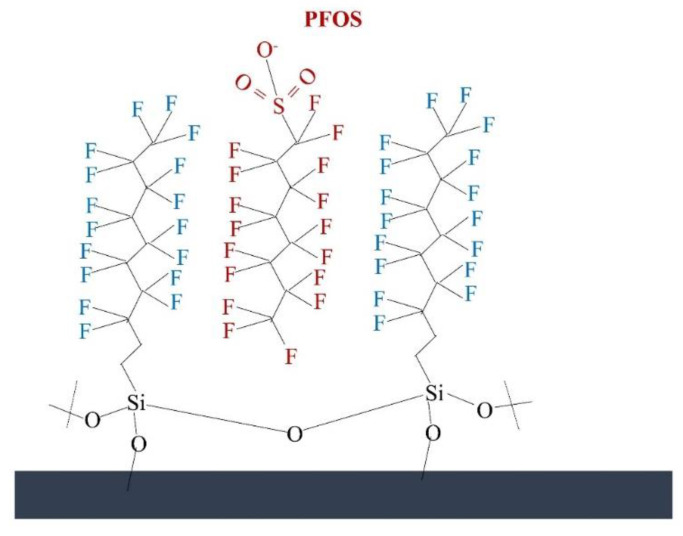
PFAS binding in the modified surface.

Linear fluorinated silane-functionalized aluminum oxide hydroxide-modified membranes with an effective pore size of 1 µm were fabricated by Johnson et al. [115]. The removal mechanism of PFOA (0.39 ng/L) and PFOS (0.86 ng/L) in this work was based on the hypothesis that the perfluorinated side chains present on the prepared surface would have a favorable fluorophilic (C−F···F−C) interaction with PFAS (Figure 4). The fabricated membrane (containing 13–17 fluorine atoms) efficiently removed >90% (unmodified membrane showed ~80% removal) of the PFOA and PFOS at a high flux rate (1223 LMH, pressure drop 0.0413–0.317 bar, pH 7.5, filtration time 30 min). This work shows the advantage of using a hydrophobic surface to remove PFAS by adsorption to the membrane’s surface. As the fluorophilic interaction is strong, the membrane will eventually reach maximum capacity and rejection will be impacted; furthermore, such a mechanism requires regeneration when reaching adsorption capacity. These have not been addressed in the presented work. Furthermore, hydrophobic membranes have a higher fouling tendency in comparison to hydrophobic membranes, but fouling of the membrane is not addressed. However, due to the hydrophobic surface, a high-pressure drop (0.317 bar) was observed across the membrane thickness during the operation. To overcome or reduce the back pressure drop, the membrane surface was further modified with hydrophilic poly(ethylene glycol) (PEG) units. The introduction of further PEG modification successfully reduced the pressure drop (~0.0413 bar), and 99.9% PFAS removal was achieved. Furthermore, a detailed analysis of membrane-fouling and regeneration/lifespan of the modified membrane needs to be done to understand the process in detail [115].

In addition, amyloid fibril-based membranes were used for the removal of 16 different PFAS (~400 ng/L) from wastewater [28]. The membranes were able to efficiently remove 99% of long- and medium-chain PFAS (Molecular weight 214–714 Da). Solution pH was shown to impact the removal efficiency, as at low pH values (about pH of 2) the amyloid fibril membrane becomes more positively charged and attracts negatively charged PFAS (due to electrostatic interactions). Furthermore, the significant role of hydrophobic interaction between PFAS and amyloid surface was also shown with long-chain PFAS, which were adsorbed to the hydrophobic surface. Finally, it was suggested that the amyloid-carbon hybrid membrane (permeability ~1739 LMH/bar) shows better performance in short-chain PFAS removal (>96%) compared to a pristine NF membrane (20–90%), resulting from a highly adsorbent amyloid-carbon surface. The operating cost of this membrane ($ 0.042/m^3^, energy requirement 0.2 kWh/m^3^) is moderate compared to the NF membrane ($ 0.016–0.16/m^3^ [38], energy requirement 0.528 kWh/m^3^), and it is made up of by-products from the dairy industry (prepared in the water phase and biodegradable), making it suitable, appropriate, and convenient for such an application. While results are encouraging in terms of an economical and sustainable treatment approach that can be implemented to remove PFAS efficiently from wastewater, further consideration regarding scaling up, fouling, and operational conditions is necessary [28].

### 4.2. Ceramic Membranes

In addition to polymeric membranes, ceramic membranes have been used to remove PFAS. The principal advantages of inorganic ceramic membranes over polymeric membranes include high thermal stability, mechanical strength, and chemical stability. Ceramic membranes can withstand a broad range of temperatures and harsh pH environments. Furthermore, they typically do not exhibit irreversible changes in structure that may affect their operational performance [116,117,118]. Commonly used materials to develop ceramic membranes are microporous glasses, titania, silica, alumina, zeolites, and zirconia [119]. Methods used to fabricate inorganic ceramic membranes mainly involve sol-gel, solid-state sintering, chemical extraction, phase-separation, and chemical vapor deposition [116]. The pore size of commercially available ceramic membranes ranges from approximately 4 nm to 10 µm, which is similar to MF, UF, and NF membranes [120]. Therefore, ceramic membranes are less effective in rejecting and removing most PFAS, especially when addressing short-chain PFAS.

While some ceramic membranes are not able to directly remove PFAS, they can be used for coupled processes, which include adsorption and filtration, mainly used to separate the adsorbing material from the liquid phase; for example, Murray et al. (2019) [25] used a commercial ceramic membrane along with a superfine powder activated carbon adsorbent to remove a mixture of 12 different types of PFAS collected from a firefighting training area, with a concentration ranging between 1.18–55.7 ng/L. In addition to removing the activated carbon (20 L adsorption tank, 100–500 mg/L), the ceramic membrane (60–65 LMH, crossflow velocity 0.19 m/s, permeate flow 43–48 mL/min, experiment duration 42–200 h) was able to mainly remove long-chain PFAS. The adsorption of PFAS was achieved by using different adsorbents (granular activated carbon (GAC) and super fine powder activated carbon (SPAC)), and the parameters affecting the adsorption capacity (SPAC > 480 × GAC) were adsorbent particle size (SPAC—0.88 µm, GAC—650 µm), adsorbent pore size (SPAC—2.70 nm, GAC—2.58 nm), adsorbent specific surface area (SPAC—927 m^2^/g, GAC—784 m^2^/g), and chain length of PFAS (C_4_–C_8_).

Resulting from adsorption followed by ceramic nanofiltration, the permeate sample contains substituted perfluoroalkane derivatives and sulfonamide precursor substances (such as long-chain (3.3% FPeSA (C_5_) and 0.7% FOSA (C_8_)) and short-chain(43% FBSA (C_4_) and 53% FPrSA (C_3_)), which indicates the inefficient outcome of the system. The pressure drop during the operation (reflected in specific flux calculation) confirms membrane fouling, mainly by the formation of a cake layer (after ~150 L of wastewater treatment). During the activated carbon separation process, the carbon particles can build up on the membrane surface and serve as a narrow protective layer that inhibits the foulants from reaching the membrane surface. It is possible that the foulants are getting adsorbed and removed by the protective layer of carbon formed on the membrane surface, which leads to a long-term operation of the membrane with minimum fouling. For the long-term operation, the authors have also used back-pulses for 2 s in every 5 min of interval to prevent fouling. Further effort is needed to reduce the operating cost and remove short-chain PFAS [25].

### 4.3. Polyamide-Modified Thin Film Composite Membranes

**Figure 5:**
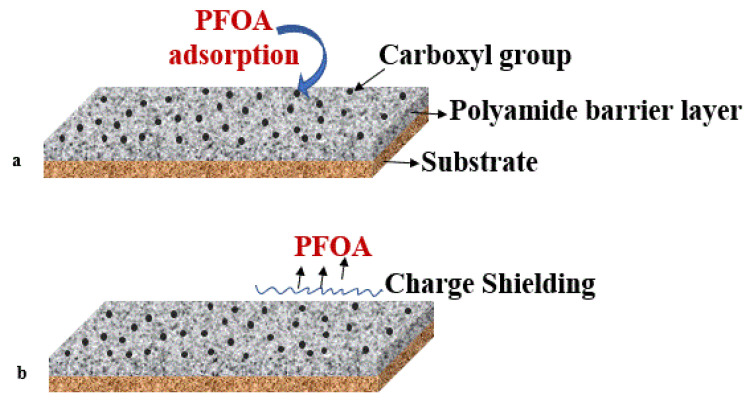
Polyamide barrier layer on membrane surface with (**a**) PFOA adsorption and (**b**) charge-shielding by carboxyl groups preventing PFAS transport to the membrane surface.

Another approach to modifying membranes to enhance PFAS rejection was suggested by Nadagouda and Lee (2021); they suggested modifying the NF/RO membrane’s surface charge using nano-porous polyamide. This modification increased the negative charge at the membrane’s surface. A negative charge was estimated to enhance the rejection of anionic PFAS molecules by electrostatic repulsion between the negatively charged membrane and anionic PFAS (Figure 5a,b), which can also help prevent fouling. Moreover, the authors implied that elevated pH would improve the rejection, as carboxyl groups at the membrane’s surface may be deprotonated, and therefore, the membrane’s negative charge will increase [121].

### 4.4. Modified Silica Membrane

Additional research by Zhou et al. (2016) [122] shows how PFAS (PFHxS, PFOS, PFHpA, PFOA, PFNA, PFDA, PFUnDA, PFDoDA, and PFTA, concentration 0.5–50 ng/L, pH 3, volume 1 L) removal efficiency can be enhanced by a novel Fe_3_O_4_ nanoparticle-coated silica membrane with fluorinated groups (Fe_3_O_4_@SiO_2_-NH_2_&F_13_). PFAS could be adsorbed by the magnetic Fe_3_O_4_ particles due to electrostatic and F-F interaction (range of adsorption capacity is 13.2–111.14 mg/g, at room temperature for 24 h). Compared to activated carbon (58.61% adsorption), the magnetic material (86.29% adsorption) showed better adsorption capacity. The authors confirm the stability and reusability (5 cycles of adsorption and regeneration, by applying an external magnetic field and washing with acetonitrile: ammonia-methanol (7 mol/L) (6:4 *v*/*v*) and ethanol: water (5:5 *v*/*v*) solution three times, respectively) of the material, and showed no significant reduction (<5%) in efficiency even after the fifth run. Furthermore, the addition of organic matter, i.e., humic acid, did not show a significant influence on rejection; however, additional information is needed on fouling and fouling mitigation to understand the applicability of such membranes [122].

### 4.5. Graphene Oxide (GO)-Nanofiltration-Membranes

GO-nanofiltration-membranes were developed by Meragawi et al. (2020) [123] to remove PFAS from wastewater, and they exhibited inferior performance (74.3% efficiency for 50 ppm PFOA, transmembrane pressure 1 bar, permeate flow rate 10 ± 2.1 LMH/bar) compared to normal NF membranes [96]. GO had an extended interlayer spacing in aqueous media because of water molecules clustering around the oxidized functional groups. This expanded interlayer spacing allows water transport but prohibits PFOA molecules from passing the membrane due to size exclusion.

**Figure 6:**
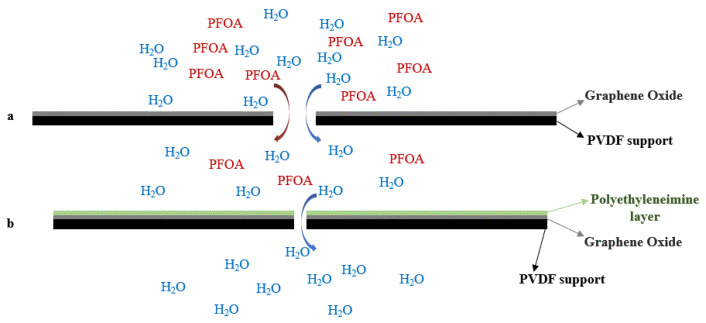
Schematic diagram of water permeance and retention changes for Graphene Oxide (GO) and GO-PEI membranes. (**a**) GO layer on top of PVDF support. (**b**) PEI layer on top of GO layer (interlayer space reduction).

Further surface modification of the GO-membranes by solution casting of polyethyleneimine (PEI) improved its permeance, selectivity, and mechanical stability. The electron-rich polyethyleneimine deoxygenates GO, leading to a reduction in interlayered spacing and improving the hydrophobicity of the surface layer. Furthermore, the retention of GO-PEI-modified membranes showed improved performance, resulting from the enhanced steric exclusion derived from the decreased interlayer spacing (Figure 6a,b). The introduction of polyethyleneimine underwent reduction and cross-linking reactions, and demonstrated a performance improvement (96.5% removal, permeate flowrate 15.9 ± 1.3 LMH/bar, which is an improvement of >22%) for the same concentration of PFOA. Furthermore, antifouling property and better abrasion resistance were observed due to the hydrophilic surface (contact angle 24.7° of compared to GO membrane with a contact angle of 54.8°). Antifouling ability was evaluated while filtering a solution containing bovine serum albumin (BSA, 30 mg/L, for 2 h). The fouled GO-PEI membranes showed promising results with sodium hydroxide (at pH 9 with 50 mL for 15 min, followed by DI water wash for 2 h) wash instead of ethanol (at pH 9 with 50 mL for 15 min, followed by DI water wash for 2 h) wash. This work demonstrates that the hydrophilic GO-PEI surface improves water permeance and shows that incorporating a hydrophilic PEI layer on the top of the GO layer lowers the requirement of energy for water permeance into hydrophobic pores, leading to further improvement while also improving PFOA rejection as a result of membrane pore size [123].

### 4.6. Metal Organic Framework (MOF)-Based Membranes

In recent years, metal organic frameworks (MOFs) have been shown to be efficient for wastewater treatment and adsorption processes [124,125], resulting from their unique properties, including their large surface area, high pore volume, high adsorption capacity, and high conductivity.

**Figure 7:**
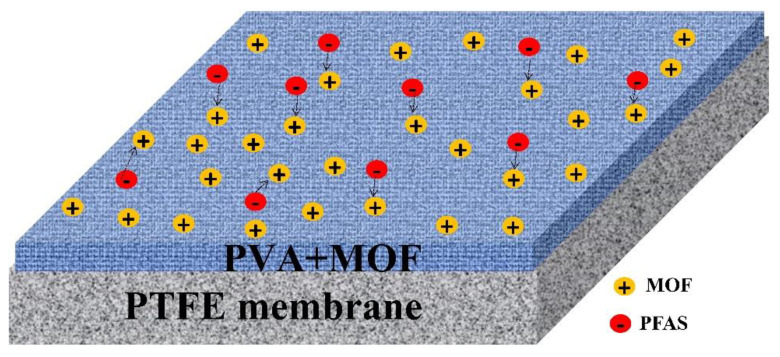
Metal-Organic Framework (MOF) modified PTFE membrane.

A MOF integrated dual-layer membrane (Figure 7) was employed to remove ammonia and PFAS (PFOS, PFOA, PFBS, PFPeS, PFHpS, PFPeA, PFHxA, PFHpA, and PFHxS) from landfill leachate. Zhang et al. (2022) [126] used a dual-layer membrane with a hydrophilic upper surface coating a hydrophobic membrane (PTFE) for membrane distillation and pervaporation. The hydrophilic layer of aluminum fumarate-based MOF mixed with Polyvinyl Alcohol (PVA) was coated on top of the conventional hydrophobic PTFE membrane to overcome the low separation rate of PFAS and the pore wetting encountered during the distillation process while using conventional pristine PTFE membranes. The authors assessed the removal efficiency of pristine PTFE membranes and modified PTFE membranes with different PVA to MOF ratios (100:0 (PSA0), 100:1 (PSA1), 100:5 (PSA5), and 100:10 (PSA10)). The hydrophobic/hydrophilic dual-layer incorporated with MOFs showed a combined effect of membrane distillation and pervaporation to remove PFAS. The rejection of total PFAS increased (91.4 to 98.4%) when the MOFs loading was increased (0 to 5 wt%). Further increment of MOF loading (to 10 wt%) had a limited impact on PFAS rejection, probably from MOF particles agglomeration, which reduces the active surface area.

**Figure 8:**
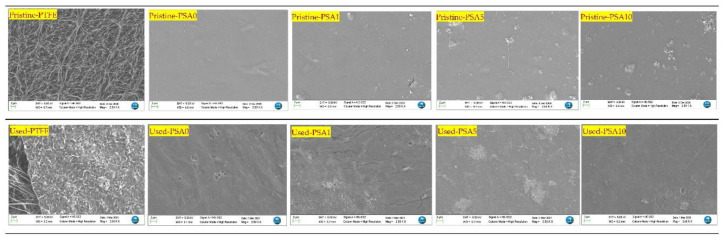
Pristine and used membranes after PFAS treatment (reprinted with permission from Ref. [126]. Copyright 2022 Elsevier).

Overall, the optimum loading of MOFs was found to be 5 wt%. The changes in the surface morphology of the pristine and used membranes clearly define the fouling (Figure 8) and confirm that the modified membrane has a better rejection compared to unmodified PTFE membranes. The used PTFE membrane showed higher fouling agents (from EDX analysis) compared to modified membranes in most of the cases (PTFE: Al 2.2%, Ca 1.6%, Si 6.3%, Mg 0.6%, Na 1.4%; PSA0: Al 0.2%, Si 0.8%, Mg 0.2%, Na 0.8%; PSA1: Al 0.3%, Si 1.4%; PSA5: Al 0.1%, Si 0.4%, Na 0.4%; PSA10: Al 0.1%, Si 0.6%, Na 1.7%). Finally, the fouling of MOF-modified membranes questions the stability and reusability of such membranes, which needs further assessment.

### 4.7. Functionalized-MXene Hollow Fiber Membranes

**Figure 9:**
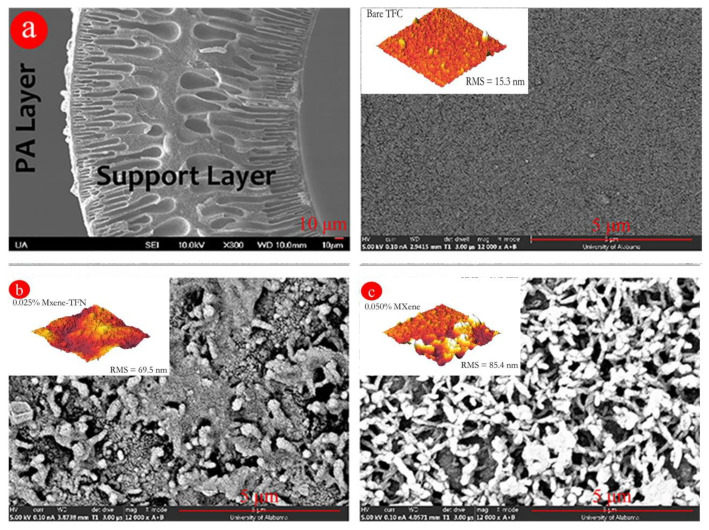
SEM and AFM images of (**a**) bare thin film composite membrane (Cross-section (left) and top surface (right)), (**b**) 0.025 MXene−Membrane, and (**c**) 0.050 MXene—Membrane (reprinted with permission from Ref. [112]. Copyright 2022 ACS).

Functionalized-MXene (Ti_2_C_3_T_x_, 0–0.05 wt% MXene) thin-film nanocomposite hollow fiber membranes (Figure 9) were successfully developed by Le et al, for PFOS removal from water. The thin film was formed through interfacial polymerization [112], and the coated polyamide-MXene layer (optimum at 0.025 wt% MXene) on polysulfone hollow fiber support was able to increase PFOS rejection from 72 to 96% in comparison to a PES polyamide hollow fiber. Furthermore, the addition of an MXene layer increased the permeability from 13.9–29.26 LMH/bar.

Factors considered to influence PFOS rejection following modification were mainly electrostatic interaction and size exclusion. Due to the lamella shape and interlayer of MXenes inside the polyamide layer, a different transport mechanism was observed for ions (interlayer spaces formed due to lamellar structure by MXenes inside the polyamide layer results in swelling when exposed to water while filtration takes place, which promotes passing of MgSO_4_ ions (0.35, 0.23 nm)), water (flowing through intralayer channels and nanosheet gaps), and PFOS molecules (prevent the transport by MXene channels). According to the suggested transport mechanism, water passes through the membrane by incorporating diffusion through the PA layer as well as the Mxene intralayer channels, resulting in enhancing the membrane permeability and rejection of PFOS. The stability of the MXene-modified membrane was tested under static (no pressure, 3 months) and dynamic (4.48 bar, 24 h) conditions, which confirms there was no significant leaching of the MXene particles. The authors also confirm that membrane–PFOS interaction is reversible, while for the unmodified membrane it was based on irreversible adsorption, leading to partial pore blocking.

To summarize, due to their relatively easy fabrication approaches and low cost, most of the membranes used for PFAS removal are polymeric, but a few of the recently reported work shows that silica and ceramic membranes are also employed for PFAS removal from wastewater [25,121]. Several aspects of these research papers are tabulated below (Table 3).

From Table 3, it seems as though there is no direct connection between the bulk materials (e.g., polymeric or ceramic) used for fabrication and PFAS removal efficiency, and that the rejection is mainly controlled by the active layer and its specific physiochemical properties (e.g., hydrophobicity and charge). In addition to the active layer properties, membrane pore size and porosity play a critical role in PFAS rejection, and membranes with larger pores were shown to be inefficient in PFAS removal. On the other hand, modification of the membrane’s surface properties might not always favor fouling mitigation, which could significantly impact the lifetime of the membrane and require frequent cleaning.

## 5. Coupled Membrane Technology

Physical separation techniques (e.g., adsorption, ion-exchange resins, membrane separation, e.g., NF or RO) are able to remove PFAS from the liquid phase onto adsorbent materials or into a concentrated brine solution. As these processes are unable to destroy PFAS, which are considered ‘forever chemicals’ [63,127,128] resulting from the strong C–F bond, there is a need to deal with the disposal of absorbents contaminated with PFAS or PFAS-concentrated-brine which may raise secondary pollution risks.

Complete degradation technologies (Details given in SI 1) for PFAS are mainly based on high-energy incineration or advanced oxidation processes, including microwave thermal treatment, electrochemical oxidation, photocatalytic degradation, sonochemistry, and pyrolysis. These novel and extreme PFAS degradation techniques are expensive, especially when dealing with the large volume and high flow rate of water containing PFAS. Thus, it is ideal to utilize other relatively low-cost technologies to reduce PFAS wastewater volume first and concentrate PFAS along with co-contaminants. The wastewater containing highly concentrated PFAS may be transferred to a disposal well deep underground [129,130] or a PFAS-specialized degradation plant for complete destruction. This approach is also expensive, requires further treatment steps, and does not always eliminate the concentrated PFAS streams [63,131].

To overcome these limitations, membrane separation was shown to be coupled with PFAS destruction through electrooxidation, photocatalysis, and more. This approach allows the direct breakdown of PFAS at the membrane’s surface while overcoming typical limitations of such processes, including insufficient contact area or diffusion limitations [132,133].

The next section includes some examples of novel destructive approaches coupled with membrane separation.

### 5.1. Electromagnetic Ceramic Membrane

**Figure 10:**
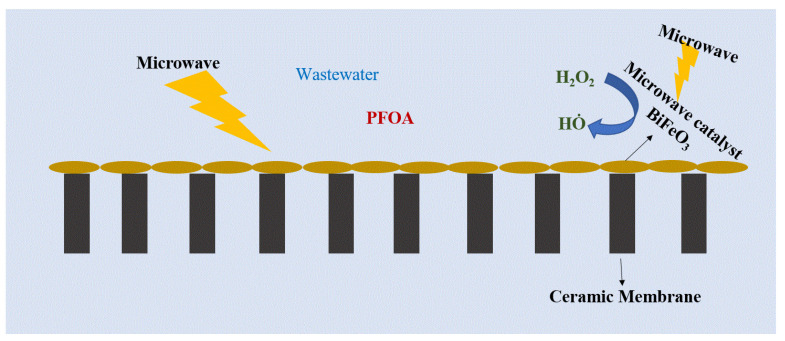
Schematic representation of the microwave catalyst grafted ceramic membrane for PFOA removal from wastewater.

**Figure 11:**
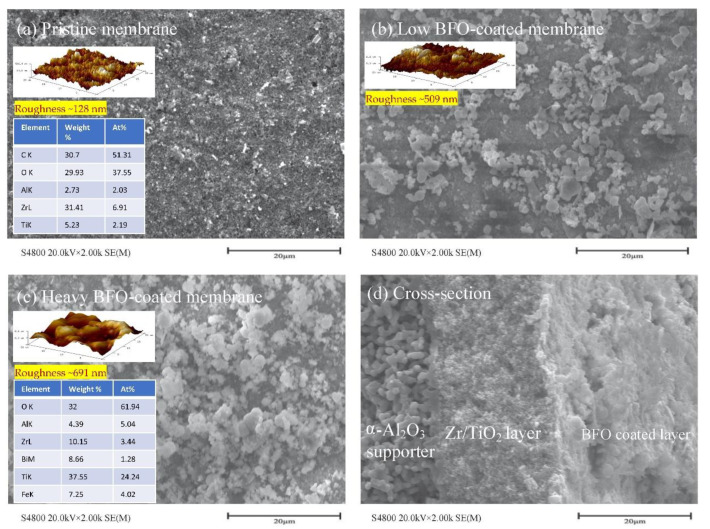
SEM and AFM images of (**a**) pristine (with elemental analysis), (**b**) low BFO-coated, and (**c**) heavy BFO-coated (with elemental analysis); and only SEM image of (**d**) cross-sectional of BFO coated membranes (adapted from Ref. [134]).

BiFeO_3_ (BFO) catalyst-coated ceramic membranes (140 nm pore size, catalyst suspension loading 4–7 ng/L) were tested under a microwave environment (7.2 W cm^−2^) to achieve a Fenton-like reaction (Figure 10) [134]. The surface morphology of pristine and low and heavily coated BFO membranes (SEM, elemental analysis, and AFM) is shown in Figure 11. According to Liu et al. [134], the pristine membrane shows only 2% removal of PFOA (25 µg/L initial concentration), whereas the modified membrane drastically improves the efficiency to 65.9% while H_2_O_2_ was additionally added to the system within 2 min of hydraulic time (permeate flowrate 43 LMH, pressure 0.42–0.96 bar, and power density 416–472 Wm^−3^). Even though the BiFeO_3_-coated membrane blocks ~20% of the membrane pores, the author claimed that microwave irradiation improves productivity and permeates flux. The removal/destruction mechanisms are described in terms of several stages. First, PFOA is adsorbed on the membrane and catalyst surface, followed by full penetration to the membrane filter after reaching adsorption equilibrium. Next, PFOA is destroyed by the formation of hydroxyl radicals by a Fenton-like reaction. Furthermore, the increase in transmembrane pressure (from 0.42–0.96 bar) confirms some fouling, probably due to degraded by-products adsorbed on the membrane’s surface. However, further investigation is necessary to understand the scalability and applicability of electromagnetic ceramic membranes for PFAS removal [134].

### 5.2. Reactive Electrochemical Membrane

A recent study by Le et al. (2019) [135] showed the use of a ceramic Ti_4_O_7_ Reactive Electrochemical Membrane for electrochemical oxidation of PFOA and PFOS (pH 7). The Reactive Electrochemical Membrane operated at a high flux of 240 LMH with a residence time of 11.3 s, resulting in almost complete removal of PFOS and PFOA at open circuit potential of 3.3 and 3.6 V/SHE, respectively. The membrane served as an anode. At first, the PFOA forms a perfluorinated alkyl radical via direct electron transfer, and then it undergoes Kolbe decarboxylation and produces C_7_F^•^_15_ radicals. These radicals react with ȮH to form C_7_F_15_OH while eliminating HF, and at the last stage it forms PFHpA by hydrolysis. The problem with such techniques is that they generate a shorter chain PFAS with similar toxicity.

In terms of energy requirements, 5.1 kWhm^−3^ were required to treat 10 µM PFOA and 6.7 kWhm^−3^ for PFOS. Treatment did not totally destroy the PFAS but reduced it to safe levels for drinking water (PFOA final concentration: 86 ng/L, PFOS final concentration: 35 ng/L).

This process implies high degradation efficiencies at a relatively low operating cost in comparison to other existing technologies (photocatalysis, microwave-hydrothermal, ultrasonication, etc.); further details and comparison is given in the next section. While results are highly encouraging, implementation of this technology in real-life applications requires investigating the performance of Reactive Electrochemical Membranes with actual industrial wastewater. Additionally required is the understanding of the complete breakdown or destruction of the parent compound as well as by-products. Furthermore, the use of multiple Reactive Electrochemical Membranes in a series can help remove the possible intermediate/by-products formed during the oxidation of PFAS [135].

Another study by Zhuo et al. (2012) reported effective removal of PFOA (~97.5%) using boron-doped diamond (BDD). While BDD was shown to be efficient in PFAS destruction, the cost of the BDD electrode is extremely high (~$ 7000/ m^2^), which makes it unrealistic in real-life applications [136].

### 5.3. Phosphorene Nanocomposite Membranes

**Figure 12:**
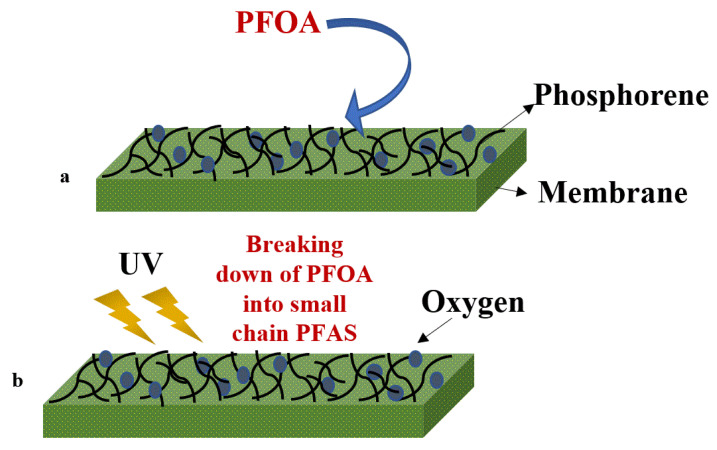
Schematics of the filtration process of PFOA on phosphorene membranes (**a**) followed by either treatment using UV light or liquid aerobic oxidation (**b**).

**Figure 13:**
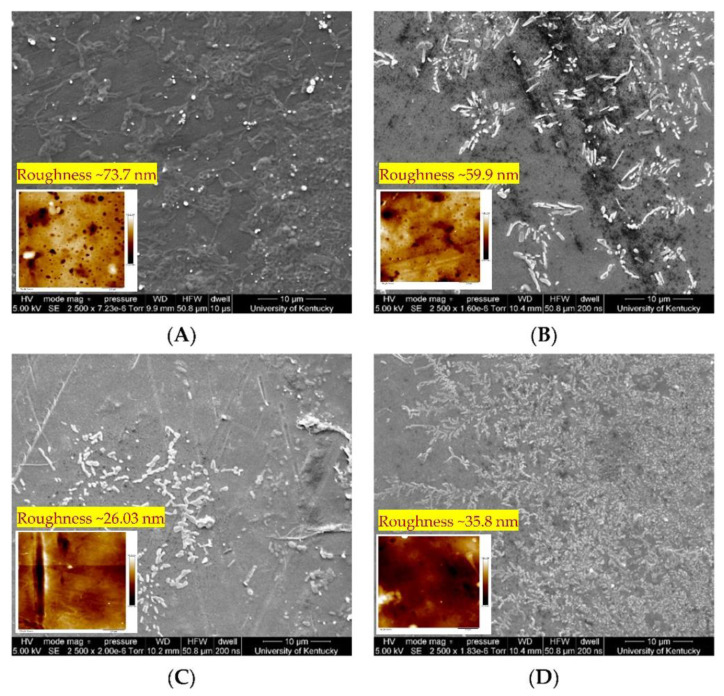
SEM and AFM images of membrane surface: (**A**) plain/clean membrane, (**B**) the membrane after PFOA filtration, (**C**) the membrane after PFOA filtration and irradiation with UV, and (**D**) the membrane after PFOA filtration and oxygenation (adapted from Ref. [113]).

Eke et al. [113] developed a dual-function phosphorene nanocomposite membrane for filtration combined with treatment by UV irradiation (365 nm, 200 min operation) or by oxygenation (at a flowrate of 3 L/min for 280 min) (Figure 12a,b). Perfluorooctanoic acid (PFOA, 100 ppm) was removed using a nanohybrid membrane made of sulfonated polyether ether ketone and phosphorene. The low bandgap of phosphorene on the membrane surface provides electronic and photocatalytic properties, which simultaneously helps to remove as well as destroy the PFOA from the membrane surface. The surface morphology of this membrane is shown in Figure 13 (SEM and AFM images of the pristine membrane after PFOA removal and after UV and oxygen treatment on membrane surface). The SEM images strongly suggest (Figure 13) that the surface of the membrane has changed after the UV/oxygen treatment, but further information is required to confirm the degradation of the membrane surface. However, XPS analysis of the membrane surface suggested that the fluorine content on the membrane surface was low after UV treatment (to some extent), whereas oxygen treatment was not significantly impactful. The stability of the membrane was also analyzed by the authors, implying that the phosphorene leaching was <1% of the initial phosphorene added to the membrane surface. Almost complete rejection was achieved for PFOA, whereas the recovery of flux for reverse-flow filtration was 84%, indicating there was no significant attachment of PFOA on the membrane surface (at 2.06 bar and room temperature, flux varies between ~123–145 LMH). The small amount of PFOA accumulated at the membrane’s surface during the treatment process was destroyed by UV light (98.4%) and liquid oxygen (96.6%). This study highlights the removal and destruction of fluorinated compounds from wastewater, but the long-term impact of photocatalysis on membrane degradation and stability is not addressed [113].

Additional reported work on combined technology such as membrane-ion exchange resins, membrane-adsorption, membrane UV/O_2_, membrane-photocatalysis, and membrane-electrocatalysis for PFAS wastewater treatment is summarized in Table 4.

According to Table 4, membrane separation process can be used first to reduce PFAS contaminated wastewater volume and concentrate PFAS into its highest possible concentration, and then other technologies can be used to degrade the PFAS completely. For instance, a study by Boonya-Atichart et al. (2017) [46] discovered that the NF membrane efficiently removed >99% PFOA from wastewater; next, the concentrated PFOA solution was subjected to photocatalytic degradation, which showed ~60% destruction. In the case of destruction technologies, it is always necessary to find an economical, efficient, and suitable process. A brief discussion about cost analysis is provided in the next section. A few of the conventional and modified membranes used for PFAS removal and destruction are summarized in Table 5.

## 6. Cost Analysis of the PFAS Treatment Technologies

Overall, PFAS treatment technologies are energy intensive and expensive. Although the treatment technologies for PFAS are rapidly improving, only a few of them are currently mature enough to be deployable for full-scale operation. In particular, adsorption by activated carbon and ion exchange resins as well as separation by membrane technology have proven to be the most effective and practical methods for PFAS-contaminated water treatment.

The treatment cost of wastewater containing PFAS largely depends on the treatment goals and discharge requirements, which are mostly based on the guidelines established by government regulations. Additionally, the amount of wastewater can also influence the treatment cost. The treatment cost of a large volume of contaminated water can cost less in comparison to a small volume, since the fixed cost is constant and almost the same while a large amount of wastewater is being treated [33,38].

The key cost factors to consider for PFAS treatment technology include: (a) presence of co-pollutants in the system, (b) PFAS species, (c) influent concentration, (d) contact time between adsorbent and pollutant, (e) adsorbent or membrane regeneration, (f) adsorbent or membrane reusability, (g) adsorbent or membrane lifecycle, (h) adsorbent disposal, (i) pre-treatment of the effluent, and (j) energy and concentrate disposal for membrane technology. Generally, membrane technology is more expensive than the adsorbent process for the removal of PFAS. The cost evaluation of various processes is tabulated below (Table 6).

From the above Table 6, most of the effective techniques are expensive. Hence, it is necessary to develop an eco-friendly, novel, efficient, and economical technique for PFAS which can be implemented easily to overcome this global issue. There are several possible approaches that can be implemented efficiently in the future, which are discussed in the next section [33,38].

## 7. Future Directions

Currently, the available membrane treatment processes for PFAS are still dependent on conventional techniques which are incapable of destroying PFAS (e.g., RO, NF, adsorption), resulting in a concentrated stream that needs to be disposed of or destroyed safely. Therefore, future technologies should address PFAS destruction coupled with separation.

A few possible approaches for efficient, economical, and environment-friendly ways to remove PFAS are enlisted below:

Thin-film nanocomposite membranes are reportedly used as NF or RO membranes for monovalent and divalent separation. The surface modification of these membranes using graphene oxide, graphitic-carbon nitride, Mxenes, COF, etc., can improve the membrane performance by severalfold. Furthermore, mixed matrix membranes (MMM) [148] can be efficient in the removal of PFAS from wastewater when the support matrix contains adsorbing materials. Based on the hydrophobic/hydrophilic nature of the membrane, the targeted short/long-chain PFAS can be removed from wastewater [148,149].

Modification of surface properties in terms of surface charge and hydrophobicity was shown to control the removal of a targeted PFAS from the wastewater matrix [150]. This is specific to the charge of the PFAS and was mainly explored with anionic PFAS such as PFOS and PFOA. Addressing the removal of cationic or zwitterionic PFAS [151] will require tailoring specific surface properties. Furthermore, short and ultra-short PFAS (C4 and below) are less impacted by hydrophobicity and will not be adsorbed to hydrophobic membranes. It should be noted that surface modification also impacts the fouling tendency of membranes; while hydrophobic membrane may remove long-chain PFAS better, the modified surface may result in organic and biological fouling, which will reduce the treatment efficiency.

Finally, coupling filtration with degradation is a promising technology for PFAS removal due to the concentration of PFAS at the membrane’s surface and low diffusion limitation. Therefore, novel nanocomposite electrodes coupled with membrane filtrations can enhance PFAS degradation by allowing (a) rapid activation through direct charge transfer followed by (b) mineralization via electrogenerated reactive oxygen species [152]. While electrochemical processes were shown efficient, similar mechanisms could be used based on other destructive methods such as photocatalysis and sonolysis [46]. 

## 8. Conclusions

PFAS are a large group of anthropogenic chemicals characterized by their high chemical stability, hydrophobicity, oleophobicity, and persistence in environmental decomposition. Resulting from high volumes of contaminated water and wastewater, membrane systems were shown to be an efficient treatment and separation approach.

This review addresses the removal of PFAS via known commercial membrane approaches such as RO and NF and presents their advantages, disadvantages, and removal mechanisms. In most cases, the membrane filtration process can remove >99% of PFAS from wastewater (regardless of the presence of other organic and inorganic impurities present in the system) and produce potable water. Furthermore, we present novel membranes—based on nanomaterials or specific surface modifications—which were shown to remove PFAS more efficiently (at the laboratory-scale). Finally, as membranes are able to reject and concentrate PFAS, there is always a need to treat the contaminated brine. Therefore, we present novel membrane systems which couple PFAS separation with ‘in-situ’ PFAS destruction.

It should be noted that most of the experimental studies have been carried out using synthetic wastewater that includes, for example, an excessive dosage of adsorbent materials, acidic pH, high concentrations of PFAS spiked in distilled water, etc. These artificial conditions do not represent either a full-scale wastewater treatment plant or real environmental conditions. Furthermore, the artificial conditions used are not representative of the possible scaling, organic fouling, and biofouling issues that could be found in a real water matrix. Overall, the presence of PFAS in water still remains a big concern since there is no single method ensuring their complete decimation; however, the use of membrane coupling ‘in situ’ destruction is a promising approach that requires further research.

## Figures and Tables

**Table 1:** Advantages and limitations of techniques for removing Per- and Polyfluoroalkyl Substances (PFAS) from wastewater.

Advantages	Limitations
***Granular activated carbon (GAC) or powder activated carbon (PAC)***	
Can remove low concentrations (ng/L) from drinking water [33] compared to other methods (UV [34], Ozone [34], modified silica [35], etc.). Long-chain PFAS (e.g., legacy PFAS as PFOA and PFOS) are efficiently (>90%) removed by GAC or PAC depending on the flow rate of the water, carbon bed depth, empty bed contact time, the temperature of the medium, and the presence of other organic matters [33,36,37]. Relatively low cost (0.093–0.12 $/m^3^) [33,38].	Inefficient for removal of short-chain PFAS due to weak (hydrophobic) interaction [36,39]. The presence of organic compounds reduces adsorption efficiency [25]. Regeneration and reuse are energy-intensive (0.78 $/kg) [40].
***Ion-exchange resin***	
Efficient for removal of anionic and long-chain PFAS (even for ng/L concentrations) [41]. Adsorption capacity is higher compared to GAC or PAC. Fast adsorption kinetics [36,42]. Operating cost is about 60% of GAC and PAC [38].	Less efficient for water containing organic or inorganic matter [38]. limited removal of short-chain PFAS (efficiency ratio PFOS _(C8)_:PFPrS _(C3)_ = 82) [43]. Requires expensive regeneration [40].
***Membrane separation***	
Effective for short-chain as well as long-chain PFAS [44]. Other organic and inorganic impurities are also removed [45]. High removal rate and efficiency (discharge goal 10–75 ng/L) [44]. Time-efficient compared to adsorption technique as no adsorption is required [38].	Fouling of membranes due to inorganic, organic, biological, and colloidal impurities may result in limited efficiency [24]. Requires brine management, which can be overcome by partnering it with a destruction process [46,47]. The energy requirement for membrane wastewater treatment is high compared to adsorption or ion exchange resin (~0.12 $/m^3^ permeate) [38].

**Table 2:** Recently reported Nanofiltration (NF) and Reverse Osmosis (RO) membranes for the treatment of PFAS.

Pollutant (Concentration, ppm)	Membrane Technology Used	Conditions	Water Matrix	Rejection	Ref.
PFOS: 0.5–1500	RO	pH 4 25 °C 200 psi 24 h	Real wastewater	>99%	[83]
Perfluorobutanoic acid (PFBA), perfluorobutane sulfonate (PFBS), perfluorooctanoic acid (PFOA), and perfluorooctane sulfonate (PFOS): 0.001	NF and RO	87–116 psi 22–28 °C pH 7.4	Tap water	95–99.9%	[101]
PFXxA: 0.0001–0.0003	RO, NF, and UF	pH 7	MilliQ water	69–99.2%	[75]
9 types of PFAS	NF	pH 6.7 18 °C 125 psi	Artificial ground water	95–99%	[96]
PFOA: 1	NF (negatively charged)	pH ~7 25 °C 100 psi	Simulated groundwater	∼90%	[102]

**Table 3:** Different membrane fabrication materials are employed for PFAS removal.

Membrane Type	Pollutant (Concentration, ppm)	Experimental Conditions	Water Matrix	Rejection	Reference
Polymeric	PFOS and PFOA: 0.00086 and 0.00039	pH 7.5 Room temperature Flux: 1223 LMH Pressure drop: 0.04–0.07 bar Time 0.5 h	DI water	>90%	[115]
	PFOA: 100	pH 7 Pressure: 2.06 bar Room temperature Flux: 123–145 LMH Time: 3.34–4.67 h	DI water	99%	[113]
	15 different PFAS (PFBA, PFPeA, PFHxA, PFHpA, PFOA, PFNA, PFDA, PFUnDA, PFDoDA, PFBS, PFHxS, PFOS, PFDS, FOSA, FTSA)	pH ~7.7 Temperature: 8.5 °C Water flow rate: 2.3 m^3^/h	Wastewater	99%	[78]
	PFOS and AFFF: 0.06 and 100	pH ~7 Temperature: 20 ± 2 °C Flux: 7–50 LMH Pressure: 4.14 bar Time: continuous operation for 13 days	DI water	>98%	[97]
Ceramic	12 different PFAS (PFPeA, PFHxA, PFHpA, PFOA, PFNA, PFPrS, PFBS, PFPeS, PFHxS, PFHpS, PFOS, and PFDS): 1.18 × 10^−6^–55.7 × 10^−6^	Flux: 60–65 LMH Time: 42–200 h	Real wastewater	~10% specific water flux	[25]
Silica membrane	9 different PFAS (PFHxS, PFOS, PFHpA, PFOA, PFNA, PFDA, PFUnDA, PFDoDA, and PFTA) 0.2 mg mL^−1^	Room temperature, Time: 24 h, pH 3	DI water and real wastewater	8.6–99.17% removal efficiency.	[122]

**Table 4:** Recently developed combined techniques for PFAS removal.

Processes	Materials/Approach	Conditions	Water Matrix	Remarks	References
Membrane-adsorption/Ion exchange resin	NF membrane (NF90-400), Granular activated carbon (Filtrasorb® 400), and anion exchange resins (Resin A600)	pH ~7.7, Temperature: 8.5 °C, Water flow rate: 2.3 m^3^/h.	Real wastewater (contains other impurities as well)	Combining the technologies worked in favor of the efficient removal of PFAS from wastewater.	[78]
Membrane-adsorption-Ion exchange resin	NF270 membrane, Granular activated carbon (Filtrasorb 400 and Norit 1240 W), and anion exchange resins (Purolite A600 and Purofine PFA694)	pH ~7.8, 8.5 °C, Pressure: 5–8 bar, Feedwater flow rate: 8 m^3^/h.	Real wastewater (32 different PFAS: 0.0001–0.0002 ppm)	This study expands knowledge of cost-efficient PFAS removal technology based on the pollutant concentration present in wastewater.	[38]
Membrane-adsorption	NF270 membrane and Granular activated carbon (Filtrasorb 300, Filtrasorb 600, and AquaCarb 1240C)	pH 6.7, 18 °C, Pressure: 1.7–9.6 bar, Permeate flow rate: 4.5–20.5 mL/min.	Artificial groundwater (PFAAs: 0.001 ppm)	This bench-scale study demonstrates the effective removal of long-chain PFAS (by adsorbents) and short-chain PFAS (by NF) from the wastewater, but further work is needed before it is implemented for large-scale application.	[96]
Membrane-adsorption	Adsorbents: Chemviron F-400 (density 440 kg/m^3^; 12 filters), Norit ROW 0.8 (density 381 kg/m^3^; 2 filters) and Norit 1240 EN.	-	Real wastewater	The combined process effectively removed >86% pollutants (present in ppt-range) from the wastewater.	[82]
Membrane-UV/O_2_	The membrane was a polymeric blend of polysulfone and poly ether ketone; oxygen flowrate 3 L/min, UV lamp intensity 365 nm	Pressure 2.06 bar, Room temperature, pH 7, Flux: 123–145 LMH, Time: 3.34–4.67 h	Synthetic wastewater (PFOA)	99% PFOA rejection.	[113]
Membrane-photocatalysis	NF membrane (2540-ACM5-TSF) and nano zero-valent iron as a photocatalyst (20–100 mg/L)	pH–11, Temperature: 2–45 °C, Feed flow rate: 1.4 m^3^/h, Flux: 70–150 LMH, Pressure: 3–41 bar	Synthetic wastewater (PFOA: 0.1 ppm)	In this coupling technology, Nanofiltration alone efficiently removed >99% PFOA, and the PFOA concentrated rejected water was photocatalytically degraded (~60%). This type of coupled technology needs more attention since it can first remove the pollutants and then destroy them successfully.	[46]
Activated carbon/Ceramic membrane	Ceramic microfiltration membrane (nominal pore size of 0.1 μm) and super-fine powder activated carbon (particle diameter < 1 μm)	Flux: 60–65 LMH, Time: 42–200 h	Real wastewater (12 different PFAS: 1.18 × 10^−6^–55.7 × 10^−6^ ppm).	~10% specific water flux	[25]
Membrane-Electrochemical technology	NF90 membrane	Pressure: 10.3–17.2 bar, Time: 10 min, crossflow velocity: 21.3 cm/s	Simulated wastewater (Hexafluoropropylene oxide dimer acid: 1 ppm)	The electrochemical treatment after membrane treatment appeared to be cost-efficient compared to direct electrochemical oxidation.	[47]
Membrane -electrochemical treatment	NF90 and NF270 membranes	Feed flow rate: 3.6 m^3^/h, Pressure: 10 bar, Temperature: 20 °C, Other ions present in the feed water (SO_4_^2−^, Cl^−^, Ca^2+^, and Na^+^ with concentrations of 321, 19.8, 172, and 24.9 ppm, respectively)	Simulated wastewater (PFHxA: 204 ppm)	Energy savings with NF90 membrane was 60–71% for 99% and 90% removal ratio.	[137]
Membrane-electrooxidation	NF90 and NF270 membranes	Flow rate: 3.2 m^3^/h, Permeability: 6.98–9.4 LMH/bar, Other ions: Na^+^ (162 ppm), SO_4_^2−^ (338 ppm); Feed volume: 10 m^3^; pressure: 10 bar; Temperature: 25 °C	Simulated wastewater (Perflurohexanoic acid: 100 ppm)	The treatment cost can be reduced further by replacing boron-doped diamond electrodes.	[26]
Membrane-electrooxidation	NF90 and BW30 membranes	Pressure: 10 bar, Crossflow velocity: 24.7 cm/s, Other salts present: NaCl and CaSO_4_	Simulated wastewater (mixture of PFOA, PFHpA, PFHxA, PFPeA, and PFBA with initial concentrations of 0.01 ppm each)	Efficiently removed PFAS to the below level set by the USEPA.	[138]

**Table 5:** Summary of membrane technologies employed to remove PFAS.

Technology	Membrane Used	Effectiveness	Remarks/(Rejection/Removal)	References
**Removal**	UF	Not effective	Works better with surface modification (10–75%).	[45]
	MD	To some extent	Not effective for short-chain PFAS (58–85%).	[24]
	NF	Highly efficient	May suffer from scale formation (~90–99%).	[78,84,96,97,102]
	RO	Highly efficient	May suffer from fouling and scale formation (>99%).	[83,98,99,100]
	FO	Not reported	-	-
	GO-nanofiltration-membrane	Reasonable	Increases membrane stability (74.3%).	[123]
	Ceramic membrane	Effective	Irreversible change on the membrane surface can reduce the performance of the membrane.	[25,116]
	Nanoparticle coated silica membrane	Highly effective	Membrane is stable and reusable (8.67–99.17%).	[122]
**Destruction**	Reactive electrochemical membrane	Highly effective	Reduction in operating cost is possible without compromising the final concentration of PFAS to the safe limit, but further work is needed with real wastewater (98.3%).	[135,139]
	Phosphorene Nanocomposite membrane	Highly effective	Destruction of fluorine compound after membrane treatment was removed by UV photolysis and liquid aerobic oxidation, which can also negatively affect the membrane surface (99%).	[113]
	Electromagnetic (microwave) membrane	Effective to some extent	Further improvement needed (65.9% degraded).	[134]

**Table 6:** Cost analysis of different PFAS removal techniques.

Processes	Materials	Treatment Cost/Energy Requirement	References
Adsorption	GAC (~$1.2–2.75/kg)	0.084–0.11 $/m^3^ wastewater for 10 ng/L treatment goal 0.021–0.025 $/m^3^ wastewater for 85 ng/L treatment goal	[33,36]
	Ion exchange resins (~$17.6–20.35/kg)	1.2–8.9 $/m^3^ wastewater for 25 ng/L discharge goal	[36]
	GAC and Ion exchange resins combined	0.84–3.28 $/m^3^ for 25 ng/L discharge goal~3.78 × 10^6^ L/day	[36]
Membrane	NF	0.016–0.16 $/m^3^ permeate	[28,38]
Membrane-Adsorption	-	~0.28 $/m^3^ for 90 ng/L discharge goal ~0.87 $/m^3^ for 25 ng/L discharge goal ~1.31 $/m^3^ for 4 ng/L discharge goal	[38,83]
Membrane-electrochemical oxidation	-	2.7–13.1$/m^3^ (High energy requirement)	[26,47,137,138]
Photocatalysis	Indium Oxides@254 nm light source	(Mostly depends on the catalyst); energy requirement 2106 KWh/m^3^, $295/m^3^, time required >11 h, ~89% removal efficiency	[140]
	Pt-TiO_2_@365 nm light source	Energy requirement 1458 KWh/m^3^, time required >7 h, 100% removal efficiency	[135]
Electron-beam	-	98% PFOA and 99.99% PFOS removal at 1500 kGy (~$295/m^3^)	[141]
Electrochemical treatment	Ti_4_O_7_ electrode (∼$0.36/m^2^) Boron doped diamond ($7000/m^2^)	5–32 KWh/m^3^ (high electrode cost and energy requirement)	[136,142]
Incineration	For regeneration of GAC or Ion exchange resins	~0.751$/kg	[143,144]
Biological treatment (cost not reported)	-	Selection of a proper biological entity, pre-treatment; additionally, the process takes a longer time, which increases the operating cost	[145,146,147]

## Data Availability

Not Applicable.

## Supplementary Materials

The following supporting information can be downloaded at: https://www.mdpi.com/article/10.3390/membranes12070662/s1, Table S1: Recently reported adsorption techniques to remove PFAS from wastewater, Table S2: Recently reported studies on ion exchange resins for PFAS removal, SI 1: Destruction techniques. References [23,46,48,53,56,58,66,128,132,135,141,142,143,144,145,146,147,153,154,155,156,157,158,159,160,161,162,163,164,165,166,167,168,169,170,171,172,173,174,175,176,177,178,179,180,181,182,183,184,185,186,187,188,189,190,191,192,193,194,195] are cited in supplementary materials.

## Abbreviations

PFAS	Per and Poly-fluoroalkyl Substances
PFHxS	Perfluorohexane sulfonate
PFOS	Perfluorooctanesulfonic acid
PFHpA	Perfluoroheptanoic acid
PFOA	Perfluorooctanoic acid
PFNA	Perfluorononanoic acid
PFDA	Perfluorodecanoic acid
PFUnDA	Perfluoroundecanoic acid
PFDoDA	Perfluorododecanoic acid
PFTA	Perfluorotetradecanoic acid
PFPeA	Perfluoropentanoate
PFHxA	Perfluorohexanoate
PFPrS	Perfluoropropane sulfonate
PFBA	Perfluorobutanoate
PFNA	Perfluorononanoic acid
FPeSA	Perfluoropentane sulfonamide
FBSA	Perfluorobutane sulfonamide
FPrSA	Perfluoropropane sulfonamide
FOSA	Perfluoroalkyl sulfonamide
FTSA	6:2 Fluorotelomer sulfonate
PFBS	Perfluorobutane sulfonate
PFPeS	Perfluoropentane sulfonate
PFHpS	Perfluoroheptane sulfonate
PFDS	Perfluorodecane sulfonate
GO	Graphene oxide
GAC	Granular activated carbon
PAC	Powder activated carbon
PMPA	Perfluoro-2-(perfluoromethoxy) propanoic acid
PEI	Polyethyleneimine
PVDF	Polyvinylidene fluoride
AFFF	Aqueous Film-Forming Foam
UF	Ultrafiltration
MF	Microfiltration
RO	Reverse Osmosis
NF	Nanofiltration
MD	Membrane Distillation
MOF	Metal Organic Frameworks
COF	Covalent Organic Frameworks
AOPs	Advanced Oxidation Processes
ARPs	Advanced Reduction Processes
UV	Ultraviolet
