# Supplementary material for: A Review on Removal and Destruction of Per- and Polyfluoroalkyl Substances (PFAS) by Novel Membranes
Source: Membranes (Basel). 2022 Jun 27;12(7):662. doi: 10.3390/membranes12070662 (PMC9325267; doi:10.3390/membranes12070662)
Supplement: Supplementary file 1 [file membranes-12-00662-s001.zip › membranes-1777409-supplementary.pdf]

membranes

MDPI

Supplementary Materials

A Review on Removal and Destruction of Per- and Polyfluoroalkyl Substances (PFAS) by Novel Membranes

Suman Das and Avner Ronen *

Zuckerberg Institute for Water Research, The Jacob Blaustein Institutes for Desert Research, Ben-Gurion University of the Negev, Sede-Boqer Campus 84990, Israel; sumand@post.bgu.ac.il
* Correspondence: avnerr@bgu.ac.il

Table S1. Recently reported adsorption techniques to remove PFAS from wastewater.

Adsorbate (μg/L)	Adsorbent Material	Experimental Conditions	Water Matrix	Efficiency/Adsorption Capacity	Ref.
PFOA and PFOS: (2.00 × 10^4–2.50 × 10^5)	Activated carbon (10 mg, 2450 m^2/g)	pH 3–10<br>175 rpm<br>48 h<br>25 °C	DI Water and actual wastewater (100 mL)	98%	[1]
PFOA and PFOS: 1.25 × 10^2–1.00 × 10^3.	Granular activated carbon (20–50 mg, 295.49 m^2/g)	pH 4–9<br>120 rpm<br>24 h<br>25 °C	DI Water	100%	[2]
PFOS: 500–3.25 × 10^5	Biochar (0.57–1 g/L, 0.5–2 mm)	pH 3–9<br>120 rpm<br>24 h<br>25 °C	DI Water	178-194 mg/g	[3]
PFAS: (20 different types)	Cyclodextrin polymer (10–60 mg/L)	pH ~6.7<br>40 rpm<br>48 h<br>23 °C	Milli-Q water and groundwater (10 mL)	~100%	[4]
PFOA: 5000–20 × 10^5	Carbon-polysulfide (2–40 g)	pH 2–11<br>250 rpm<br>1 h<br>25 °C<br>Flow Rate 200mL/min	DI water and surface water(5–10000 mL)	500 μg/g	[5]
PFOA: 2 × 10^5–4.5 × 10^5	Fe_3O_4@GAC (0.05 g, 2.27 nm, 687 m^2/g)	pH 2–11<br>200 rpm<br>500 h<br>25 °C	DI water (100 mL)	91%	[6]
PFOA and PFOS: 1 × 10^5	All-silica Beta-Zeolite (5 mg)	24 h<br>25 °C	DI water (5 mL)	PFOA ~ 45 mg/g, PFOS ~ 52 mg/g	[7]
PFOS: (15 × 10^3–150 × 10^3	high-silica zeolite NaY (0.1 g, 700–780 m^2/g)	pH 7.2<br>150 rpm<br>30 °C	DI water (100 mL)	120 mg/g	[8]
PFOA and PFOS: 5 x 10^5	UiO-66 and UiO-66-(F4) MOFs (1 mg/mL)	pH 3–10<br>1 h<br>150 rpm<br>25 °C	DI water	470 mg/g	[9]

PFOA	MIL-101(Cr)-MOFs (4 mg)	pH 3–10<br>12 h<br>150 rpm<br>23 °C	DI water (40 mL)	1.89 mmol/g	[10]
PFAS: 200–5 × 10^4	[NH2]–COF (100 mg/L, 1900 ± 1% m^2/g)	pH 3–9<br>0.5 h<br>500 rpm<br>23 °C	DI Water	>90%	[11]
PFAS: 8.33–133	Ferric nanoparticles (40 μg/L)	pH 3–7.5, Ozone bubbling times 15–45 s	DI Water	44%	[12]

Table S2. Recently reported studies on ion exchange resins for PFAS removal.

Adsorbate (μg/L)	Adsorbent Material	Experimental Conditions	Water Matrix	Efficiency/ Adsorption Capacity	Ref.
PFOA and PFOS: 0.1–50	Purolite A860 (capacity 0.8 meq /mL, a median pore diameter of 7.2 nm and average resin diameter of 750 μm, doseage: 50 mg/L)	pH 3–10<br>24 h<br>150 rpm<br>23 °C	Milli-Q water, and natural wastewater	50 ng/mg	[13]
PFAS: 25 × 10^3–4 × 10^5	Polyacrylic-DVB gel IRA67 (Exchange capacity = 1.6 meq/ mL; Effective size = 0.3–1.2 mm, doseage: 50 mg/L)	pH 3<br>48 h<br>150 rpm<br>25 °C	Actual wastewater	2397 mg/g	[14]
PFOA: 1.2 × 10^5	Polyacrylic-DVB gel IRA67 (Exchange capacity = 1.6 meq/ mL; Effective size = 0.3–1.2 mm, dosage: 100mg/L)	pH 4<br>48 h<br>170 rpm<br>25 °C	Actual wastewater	1167 mg/g	[15]
PFOA: 5 × 10^4–4 × 10^5	Polystyrene-DVB macroporous material IRA910 (Exchange capacity = 1.0 meq/ mL, dosage: 100mg/L)	pH 6<br>240 h<br>160 rpm<br>25 °C	DI water	1437 mg/g	[16]
PFHxS: 5 × 10^4–4 × 10^5	Polystyrene-DVB macroporous material IRA910 (Exchange capacity = 1.0 meq/ mL, dosage: 100mg/L)	pH 6<br>240 h<br>160 rpm<br>25 °C	DI water	1364 mg/g	[16]
PFOS: 25 × 10^3–4 × 10^5	Polyacrylic-DVB gel IRA67 (Exchange capacity = 1.6, dosage: 0.05 mg/L)	pH 3<br>48 h<br>150 rpm<br>25 °C	Actual wastewater	2750 mg/g	[14]
PFOS: 4 × 10^5	Polyacrylic macroporous material	pH 3<br>48 h	DI water	2000 mg/g	[17]

	IRA958 (Exchange capacity = 3.7 meq/ g; Effective size = 0.3–1.2 mm, dosage: 35-500 mg/L)	150 rpm<br>25 °C			
PFOS: 5×10^4–4×10^5	Polystyrene-DVB macroporous material IRA910 (Exchange capacity = 1.0 meq/ mL, dosage: 100 mg/L)	pH 6<br>240 h<br>160 rpm<br>25 °C	DI water	1395	[16]

SI 1. Destruction Techniques:

1.1. Biological Treatment

The biological treatment technologies for PFAS are sustainable and eco-friendly technology, which does not produce any secondary pollutants. Biological degradation of PFAS by bio-catalysts (Eg. enzymes) that directly eliminate fluorine atoms from PFAS either (a) by oxidation (addition of oxygen across the C-F bond) or (b) reduction (addition of electrons across the C-F bond). The strong C-F bond in PFAS requires a significant amount of energy to catalyze the reaction. The reported study shows that the biological treatment of PFAS can be done by microbial remediation (bacteria: *Gordonia, Acidimicrobium, etc.*), mycoremediation (fungi: Aspergillus niger, P. chrysosporium, etc.), and phytoremediation (Plant: Betula pendula, Picea abies, etc.). There are a few known microbes that can break down the C-F bond in aerobic (with oxygen) or anaerobic (without oxygen) conditions, but the degradation process is either very slow (it takes a few days to 180 days depending on the process) or less efficient compared to adsorption or membrane separation.

In the future, the defluorinating microorganisms in anaerobic digestion that can use generated methane and hydrogen as an electron donor, while PFAS act as an electron acceptor could intensify the breakdown of the C-F bond in the biodegradation of PFAS compounds. The combination of bioremediation techniques also has the potential to enhance the efficacy to degrade PFAS [18–20].

1.2. Advanced Oxidation or Reduction Processes (AOPs and ARPs)

AOPs and ARPs are capable of mineralization of PFAS in an eco-friendly way from the environment, which is one of the biggest advantages of this process. In an AOP or ARP, the efficiency of the process depends on the amount of $^{\cdot}OH$ or $O_2^{\cdot -}$ radical present in the system. Whereas the generation of $^{\cdot}OH$ or $O_2^{\cdot -}$ radicals depends on the process implemented and the operating parameters. Several treatment technologies come under AOPs and ARPs, such as ozonation [21], UV irradiation, H_2O_2 treatment, fenton, photo-fenton, photolysis, photocatalysis, and reduction with elemental iron sonolysis, electrocatalysis, and/or a combination of the above-mentioned methods. Even though these above-mentioned methods are capable of destroying PFAS efficiently, most of the processes are not suitable for the long run (reusable) or expensive, or both. Few of the most recently published AOPs and ARPs for the decomposition of PFAS shows that the time required for ~90% decomposition is a few minutes to days, which is subjected to the treatment process and operating conditions (Photocatalysis: light source, catalyst, initial pollutant concentration, etc.; Ozonation: ozone dosage, initial pollutant concentration, etc.; Fenton: catalyst dosage, pH, temperature, initial pollutant concentration, etc.; Zero valent iron: pH, initial pollutant concentration, etc.) [21–23].

Since the AOPs and ARPs are mostly costly technique, it is often used in combination with other separation techniques. In such cases, first, the PFAS contaminated water is

treated by the most popularly known efficient techniques (adsorption or membrane separation process) followed by AOPs or ARPs. This type of operation can efficiently remove and mineralize PFAS from the contaminated water, but this area of research has been poorly studied till now, which needs more attention [24].

1.3. Electrochemical Oxidation Treatment

Electrochemical oxidation is a wastewater treatment technology in which electrical current is passed through the solution to oxidize the contaminants. Electrochemical oxidation treatment of strong organic pollutants (such as PFAS) has been illustrated in several recently reported bench and pilot-scale operations [25–27].

Advantages of this process include simple, operable in the ambient environment, and does not require any chemical oxidants as additives. Though, it has a few drawbacks, which include the potential of generating toxic by-products, partial or incomplete destruction of PFAS in some cases, reduction in efficiency due to minerals build up on the anode, expensive electrodes, and possible volatilization of pollutants. In spite of these limitations, electrochemical oxidation can be a promising technology for the destruction of PFAS in certain cases because of its proven ability to mineralize PFAS with comparatively lower energy demands than thermal incineration [26,28,29].

The future work on the electrochemical oxidation of PFAS should be focused on (a) inexpensive electrodes; (b) coupling the process with another water treatment operation to destroy by-products; (c) consideration of long-term operation; (d) detailed analysis of potential by-products to understand the degradation pathway of PFAS; and (e) scale-up consideration and process parameters optimization [30–32].

1.4. Sonochemical Treatment

The sonochemical treatment process has been widely studied for environmental applications, especially for pollutant removal from wastewater due to its significant superiority, such as simple, cleanliness, energy-saving, complete destruction of pollutants without generating secondary contaminants. Numerous lab-scale reported studies on sonochemical destruction of PFAS showed outstanding potential. The ultrasound (>16 kHz) used during the sonochemical treatment generates significantly high pressure and temperature, which is sufficient to pyrolyze the PFAS present in wastewater. In the course of this process, it also generates highly reactive $^{\cdot}OH$ radical, which actively participates in pollutant degradation as an oxidizing agent [33].

The sonochemical treatment can be affected by various operating parameters, namely ultrasound frequencies, power densities, temperature, additives, atmospheric conditions, initial concentration of PFAS, chemical properties of PFAS, etc. To improve the sonochemical technology for the degradation of PFAS, the future research work can be focused on (a) reduction of the energy consumption; (b) combining this technology with other potential methods (photocatalysis, ozonation, Fenton, etc.) to increase the effectiveness and lower the energy usage; and (c) scale-up of the process [34–42].

1.5. Photocatalytic Treatment

Photocatalytic destruction of pollutants from wastewater has gained popularity for the last few decades because of its several advantages, such as eco-friendliness, simple process, complete destruction is possible, efficient, and economical. In a photocatalytic system, there are two main components involved: catalyst (Heterogeneous: TiO_2, graphene oxide, ZnO, etc.) and the light source (UV, visible, solar, LEDs, etc.). The basic working principle of this process is the generation of electron-hole pairs into the system under light irradiation on the catalyst surface. This electron-hole pair helps to produce $^{\cdot}OH$ and/or $O_2^{\cdot-}$ radicals, which react with the pollutants present in the wastewater. The photocatalytic process can effectively mineralize very small amounts of pollutants

from contaminated water, but it depends on several operating parameters (catalyst loading, initial pollutant concentration, light source, catalyst surface area, reactor geometry, pH, temperature, etc.). Photocatalysis is one of the most explored techniques for the degradation of PFAS, but the research work is mainly based on a lab-scale operation. Thus researchers are still working on the implementation of large-scale operations by overcoming the limitations (utilization of sunlight instead of UV light and large scale reactor design) of the photocatalytic process.

A distinctive and important property of the photocatalytic process is the recovery of the catalyst after the operation, which makes the catalyst reusable (without regeneration in most cases), and the process becomes economically viable. For the further improvement of PFAS removal efficiency, the combination of membrane or adsorption technology with photocatalytic processes has been reported in a few recent studies. These combined techniques have a good potential for the successful mineralization of PFAS, but this area is poorly studied which needs immediate attention [43–49].

1.6. Incineration

Incineration or thermal treatment for the destruction of toxic chemicals (PFAS) is a simple, old and effective technique, but it requires an ample amount of heat energy. This process is well known for the regeneration of contaminated adsorbents and ion exchange resins. Heat is directly applied to the PFAS-contaminated liquids (wastewater) or solids (soil, adsorbent) and vaporizes the combustion products. The vaporized products can be captured and/or subjected to further oxidation at elevated temperatures (≥1000°C). The main parameters affecting the incineration process are temperature, time, gas supply (H_2), etc. Furthermore, there are things to be careful about during the incineration process: (a) degree of destruction, (b) generation of by-products, and (c) transport of contaminants to the incineration point.

Incineration of PFAS waste may not be a cost-effective disposal method, but it ranks as the best among available technologies in the current scenario. Also, the treatment of large volumes of liquid or solid by this method is problematic and prohibitively expensive. That is why the common practice is to combine this technology with membrane technology. At first, the PFAS are separated by a membrane, and then the concentrated PFAS solution is treated by incineration [50–55].

1.7. High Energy Electron Beam

The high-energy electron beam is a nonthermal, highly efficient, chemical-free technology that uses electron accelerators to produce a huge number of highly energetic electron-mass from electricity. These highly energetic electrons participate in ionization reactions with the materials that they interact with and generate a variety of short-lived radical species which are involved in the oxidative and reductive destruction of PFAS. The efficiency of this process depends on several parameters, such as the type and thickness of the material used during the interaction, exposed time of the material to the electron beam, and beam power. This process is a promising mineralization technique to break down the short-chain PFAS, but the process needs further attention to overcome the high operating cost [56–60].

Abbreviations:

PFAS: Per and Poly-fluoroalkyl Substances.
PFHxS: Perfluorohexane sulfonate
PFOS: Perfluorooctanesulfonic acid
PFHpA: Perfluoroheptanoic acid
PFOA: Perfluorooctanoic acid
PFNA: Perfluorononanoic acid
PFDA: Perfluorodecanoic acid

PFUnDA: Perfluoroundecanoic acid
PFDoDA: Perfluorododecanoic acid
PFTA: Perfluorotetradecanoic acid
PFPeA: Perfluoropentanoate
PFHxA: Perfluorohexanoate
PFPrS: Perfluoropropane sulfonate
PFBS: Perfluorobutane sulfonate
PFPeS: Perfluoropentane sulfonate
PFHpS: Perfluoroheptane sulfonate
PFDS: Perfluorodecane sulfonate
GO: Graphene Oxide
PMPA: Perfluoro-2-(perfluoromethoxy) propanoic acid
PEI: Polyethyleneimine
PVDF: Polyvinylidene fluoride
AFFF: Aqueous Film-Forming Foam
UF: Ultrafiltration
MF: Microfiltration
RO: Reverse Osmosis
NF: Nanofiltration
MD: Membrane Distillation
MOF: Metal Organic Frameworks
COF: Covalent Organic Frameworks
AOPs: Advanced Oxidation Processes
ARPs: Advanced Reduction Processes
UV: Ultraviolet

References:

1. Deng, S.; Nie, Y.; Du, Z.; Huang, Q.; Meng, P.; Wang, B.; Huang, J.; Yu, G. Enhanced Adsorption of Perfluorooctane Sulfonate and Perfluorooctanoate by Bamboo-Derived Granular Activated Carbon. *J. Hazard. Mater.* **2015**, *282*, 150–157, doi:10.1016/j.jhazmat.2014.03.045.
2. Fagbayigbo, B.O.; Opeolu, B.O.; Fatoki, O.S.; Akenga, T.A.; Olatunji, O.S. Removal of PFOA and PFOS from Aqueous Solutions Using Activated Carbon Produced from Vitis Vinifera Leaf Litter. *Environ. Sci. Pollut. Res.* **2017**, *24*, 13107–13120, doi:10.1007/s11356-017-8912-x.
3. Hassan, M.; Liu, Y.; Naidu, R.; Du, J.; Qi, F. Adsorption of Perfluorooctane Sulfonate (PFOS) onto Metal Oxides Modified Biochar. *Environ. Technol. Innov.* **2020**, *19*, 100816, doi:10.1016/j.eti.2020.100816.
4. Wu, C.; Klemes, M.J.; Trang, B.; Dichtel, W.R.; Helbling, D.E. Exploring the Factors That Influence the Adsorption of Anionic PFAS on Conventional and Emerging Adsorbents in Aquatic Matrices. *Water Res.* **2020**, *182*, 115950, doi:10.1016/j.watres.2020.115950.
5. Lundquist, N.A.; Sweetman, M.J.; Scroggie, K.R.; Worthington, M.J.H.; Esdaile, L.J.; Alboaiji, S.F.K.; Plush, S.E.; Hayball, J.D.; Chalker, J.M. Polymer Supported Carbon for Safe and Effective Remediation of PFOA-and PFOS-Contaminated Water. *ACS Sustain. Chem. Eng.* **2019**, *7*, 11044–11049, doi:10.1021/acssuschemeng.9b01793.
6. Xu, J.; Liu, Z.; Zhao, D.; Gao, N.; Fu, X. Enhanced Adsorption of Perfluorooctanoic Acid (PFOA) from Water by Granular Activated Carbon Supported Magnetite Nanoparticles. *Sci. Total Environ.* **2020**, *723*, 137757, doi:10.1016/j.scitotenv.2020.137757.
7. Van den Bergh, M.; Krajnc, A.; Voorspoels, S.; Tavares, S.R.; Mullens, S.; Beurroies, I.; Maurin, G.; Mali, G.; De Vos, D.E. Highly Selective Removal of Perfluorinated Contaminants by Adsorption on All-Silica Zeolite Beta. *Angew. Chemie - Int. Ed.* **2020**, *59*, 14086–14090, doi:10.1002/anie.202002953.
8. Ochoa-Herrera, V.; Sierra-Alvarez, R. Removal of Perfluorinated Surfactants by Sorption onto Granular Activated Carbon, Zeolite and Sludge. *Chemosphere* **2008**, *72*, 1588–1593, doi:10.1016/j.chemosphere.2008.04.029.
9. Sini, K.; Bourgeois, D.; Idouhar, M.; Carboni, M.; Meyer, D. Metal-Organic Framework Sorbents for the Removal of Perfluorinated Compounds in an Aqueous Environment. *New J. Chem.* **2018**, *42*, 17889–17894, doi:10.1039/c8nj03312a.
10. Liu, K.; Zhang, S.; Hu, X.; Zhang, K.; Roy, A.; Yu, G. Understanding the Adsorption of PFOA on MIL-101(Cr)-Based Anionic-Exchange Metal-Organic Frameworks: Comparing DFT Calculations with Aqueous Sorption Experiments. *Environ. Sci. Technol.* **2015**, *49*, 8657–8665, doi:10.1021/acs.est.5b00802.
11. Ji, W.; Xiao, L.; Ling, Y.; Ching, C.; Matsumoto, M.; Bisbey, R.P.; Helbling, D.E.; Dichtel, W.R. Removal of GenX and Perfluorinated Alkyl Substances from Water by Amine-Functionalized Covalent Organic Frameworks. *J. Am. Chem. Soc.* **2018**, *140*, 12677–12681, doi:10.1021/jacs.8b06958.

12. Zhang, J.; Pang, H.; Gray, S.; Ma, S.; Xie, Z.; Gao, L. Pfas Removal from Wastewater by In-Situ Formed Ferric Nanoparticles: Solid Phase Loading and Removal Efficiency. *J. Environ. Chem. Eng.* **2021**, *9*, 105452, doi:10.1016/j.jece.2021.105452.
13. Dixit, F.; Barbeau, B.; Mostafavi, S.G.; Mohseni, M. PFOA and PFOS Removal by Ion Exchange for Water Reuse and Drinking Applications: Role of Organic Matter Characteristics. *Environ. Sci. Water Res. Technol.* **2019**, *5*, 1782–1795, doi:10.1039/c9ew00409b.
14. Gao, Y.; Deng, S.; Du, Z.; Liu, K.; Yu, G. Adsorptive Removal of Emerging Polyfluoroalky Substances F-53B and PFOS by Anion-Exchange Resin: A Comparative Study. *J. Hazard. Mater.* **2017**, *323*, 550–557, doi:10.1016/j.jhazmat.2016.04.069.
15. Du, Z.; Deng, S.; Chen, Y.; Wang, B.; Huang, J.; Wang, Y.; Yu, G. Removal of Perfluorinated Carboxylates from Washing Wastewater of Perfluorooctanesulfonyl Fluoride Using Activated Carbons and Resins. *J. Hazard. Mater.* **2015**, *286*, 136–143, doi:10.1016/j.jhazmat.2014.12.037.
16. Maimaiti, A.; Deng, S.; Meng, P.; Wang, W.; Wang, B.; Huang, J.; Wang, Y.; Yu, G. Competitive Adsorption of Perfluoroalkyl Substances on Anion Exchange Resins in Simulated AFFF-Impacted Groundwater. *Chem. Eng. J.* **2018**, *348*, 494–502, doi:10.1016/j.cej.2018.05.006.
17. Deng, S.; Yu, Q.; Huang, J.; Yu, G. Removal of Perfluorooctane Sulfonate from Wastewater by Anion Exchange Resins: Effects of Resin Properties and Solution Chemistry. *Water Res.* **2010**, *44*, 5188–5195, doi:10.1016/j.watres.2010.06.038.
18. Presentato, A.; Lampis, S.; Vantini, A.; Manea, F.; Daprà, F.; Zuccoli, S.; Vallini, G. On the Ability of Perfluorohexane Sulfonate (PFHxS) Bioaccumulation by Two Pseudomonas Sp. Strains Isolated from PFAS-contaminated Environmental Matrices. *Microorganisms* **2020**, *8*, 1–14, doi:10.3390/microorganisms8010092.
19. Lim, X. Can Microbes Save Us from PFAS? *ACS Cent. Sci.* **2021**, *7*, 3–6, doi:10.1021/acscentsci.1c00013.
20. Shahsavari, E.; Rouch, D.; Khudur, L.S.; Thomas, D.; Aburto-Medina, A.; Ball, A.S. Challenges and Current Status of the Biological Treatment of PFAS-Contaminated Soils. *Front. Bioeng. Biotechnol.* **2021**, *8*, 1–15, doi:10.3389/fbioe.2020.602040.
21. Franke, V.; Schäfers, M.D.; Lindberg, J.J.; Ahrens, L. Removal of Per- And Polyfluoroalkyl Substances (PFASs) from Tap Water Using Heterogeneously Catalyzed Ozonation. *Environ. Sci. Water Res. Technol.* **2019**, *5*, 1887–1896, doi:10.1039/c9ew00339h.
22. Choe, H.-S.; Kim, K.Y.; Oh, J.-E.; Kim, J.-H. Parallel Study on Removal Efficiency of Pharmaceuticals and PFASs in Advanced Water Treatment Processes: Ozonation, GAC Adsorption, and RO Processes. *Environ. Eng. Res.* **2020**, *27*, 200509–0, doi:10.4491/eer.2020.509.
23. Trojanowicz, M.; Bojanowska-Czajka, A.; Bartosiewicz, I.; Kulisa, K. Advanced Oxidation/Reduction Processes Treatment for Aqueous Perfluorooctanoate (PFOA) and Perfluorooctanesulfonate (PFOS) – A Review of Recent Advances. *Chem. Eng. J.* **2018**, *336*, 170–199, doi:10.1016/j.cej.2017.10.153.
24. Boonya-atichart, A.; Boontanon, S.K.; Boontanon, N. Study of Hybrid Membrane Filtration and Photocatalysis for Removal of Perfluorooctanoic Acid (PFOA) in Groundwater. *Water Sci. Technol.* **2017**, *2017*, 561–569, doi:10.2166/wst.2018.178.
25. Radjenovic, J.; Duinslaeger, N.; Avval, S.S.; Chaplin, B.P. Facing the Challenge of Poly- And Perfluoroalkyl Substances in Water: Is Electrochemical Oxidation the Answer? *Environ. Sci. Technol.* **2020**, *54*, 14815–14829, doi:10.1021/acs.est.0c06212.
26. Nzeribe, B.N.; Crimi, M.; Mededovic Thagard, S.; Holsen, T.M. Physico-Chemical Processes for the Treatment of Per- And Polyfluoroalkyl Substances (PFAS): A Review. *Crit. Rev. Environ. Sci. Technol.* **2019**, *49*, 866–915, doi:10.1080/10643389.2018.1542916.
27. Le, T.X.H.; Haflich, H.; Shah, A.D.; Chaplin, B.P. Energy-Efficient Electrochemical Oxidation of Perfluoroalkyl Substances Using a Ti4O7 Reactive Electrochemical Membrane Anode. *Environ. Sci. Technol. Lett.* **2019**, *6*, 504–510, doi:10.1021/acs.estlett.9b00397.
28. Chaplin, B.P. The Prospect of Electrochemical Technologies Advancing Worldwide Water Treatment. *Acc. Chem. Res.* **2019**, *52*, 596–604, doi:10.1021/acs.accounts.8b00611.
29. Schaefer, C.E.; Andaya, C.; Maizel, A.; Higgins, C.P. Assessing Continued Electrochemical Treatment of Groundwater Impacted by Aqueous Film-Forming Foams. *J. Environ. Eng.* **2019**, *145*, 06019007, doi:10.1061/(asce)ee.1943-7870.0001605.
30. Lacasa, E.; Cotillas, S.; Saez, C.; Lobato, J.; Cañizares, P.; Rodrigo, M.A. Environmental Applications of Electrochemical Technology. What Is Needed to Enable Full-Scale Applications? *Curr. Opin. Electrochem.* **2019**, *16*, 149–156, doi:10.1016/j.coelec.2019.07.002.
31. Guo, L.; Jing, Y.; Chaplin, B.P. Development and Characterization of Ultrafiltration TiO2 Magnéli Phase Reactive Electrochemical Membranes. *Environ. Sci. Technol.* **2016**, *50*, 1428–1436, doi:10.1021/acs.est.5b04366.
32. Ensch, M.; Rusinek, C.A.; Becker, M.F.; Schuelke, T. A Combined Current Density Technique for the Electrochemical Oxidation of Perfluorooctanoic Acid (PFOA) with Boron-Doped Diamond. *Water Environ. J.* **2021**, *35*, 158–165, doi:10.1111/wej.12616.
33. Pétrier, C.; Torres-Palma, R.; Combet, E.; Sarantakos, G.; Baup, S.; Pulgarin, C. Enhanced Sonochemical Degradation of Bisphenol-A by Bicarbonate Ions. *Ultrason. Sonochem.* **2010**, *17*, 111–115, doi:10.1016/j.ultsonch.2009.05.010.
34. Moriwaki, H.; Takagi, Y.; Tanaka, M.; Tsuruho, K.; Okitsu, K.; Maeda, Y. Sonochemical Decomposition of Perfluorooctane Sulfonate and Perfluorooctanoic Acid. *Environ. Sci. Technol.* **2005**, *39*, 3388–3392, doi:10.1021/es040342v.
35. Gole, V.L.; Fishgold, A.; Sierra-Alvarez, R.; Deymier, P.; Keswani, M. Treatment of Perfluorooctane Sulfonic Acid (PFOS) Using a Large-Scale Sonochemical Reactor. *Sep. Purif. Technol.* **2018**, *194*, 104–110, doi:10.1016/j.seppur.2017.11.009.
36. Campbell, T.Y.; Vecitis, C.D.; Mader, B.T.; Hoffmann, M.R. Perfluorinated Surfactant Chain-Length Effects on Sonochemical Kinetics. *J. Phys. Chem. A* **2009**, *113*, 9834–9842, doi:10.1021/jp903003w.
37. Lee, Y.C.; Chen, M.J.; Huang, C.P.; Kuo, J.; Lo, S.L. Efficient Sonochemical Degradation of Perfluorooctanoic Acid Using Periodate. *Ultrason. Sonochem.* **2016**, *31*, 499–505, doi:10.1016/j.ultsonch.2016.01.030.

38. Lin, J.C.; Lo, S.L.; Hu, C.Y.; Lee, Y.C.; Kuo, J. Enhanced Sonochemical Degradation of Perfluorooctanoic Acid by Sulfate Ions. *Ultrason. Sonochem.* **2015**, *22*, 542–547, doi:10.1016/j.ultsonch.2014.06.006.
39. Rodriguez-Freire, L.; Abad-Fernández, N.; Sierra-Alvarez, R.; Hoppe-Jones, C.; Peng, H.; Giesy, J.P.; Snyder, S.; Keswani, M. Sonochemical Degradation of Perfluorinated Chemicals in Aqueous Film-Forming Foams. *J. Hazard. Mater.* **2016**, *317*, 275–283, doi:10.1016/j.jhazmat.2016.05.078.
40. Campbell, T.; Hoffmann, M.R. Sonochemical Degradation of Perfluorinated Surfactants: Power and Multiple Frequency Effects. *Sep. Purif. Technol.* **2015**, *156*, 1019–1027, doi:10.1016/j.seppur.2015.09.053.
41. James Wood, R.; Sidnell, T.; Ross, I.; McDonough, J.; Lee, J.; Bussemaker, M.J. Ultrasonic Degradation of Perfluorooctane Sulfonic Acid (PFOS) Correlated with Sonochemical and Sonoluminescence Characterisation. *Ultrason. Sonochem.* **2020**, *68*, 105196, doi:10.1016/j.ultsonch.2020.105196.
42. Cao, H.; Zhang, W.; Wang, C.; Liang, Y. Sonochemical Degradation of Poly- and Perfluoroalkyl Substances – A Review. *Ultrason. Sonochem.* **2020**, *69*, 105245, doi:10.1016/j.ultsonch.2020.105245.
43. Tenorio, R.; Liu, J.; Xiao, X.; Maizel, A.; Higgins, C.P.; Schaefer, C.E.; Strathmann, T.J. Destruction of Per-and Polyfluoroalkyl Substances (PFASs) in Aqueous Film-Forming Foam (AFFF) with UV-Sulfite Photoreductive Treatment. *Environ. Sci. Technol.* **2020**, *54*, 6957–6967, doi:10.1021/acs.est.0c00961.
44. Li, F.; Wei, Z.; He, K.; Blaney, L.; Cheng, X.; Xu, T.; Liu, W.; Zhao, D. A Concentrate-and-Destroy Technique for Degradation of Perfluorooctanoic Acid in Water Using a New Adsorptive Photocatalyst. *Water Res.* **2020**, *185*, 1–14, doi:10.1016/j.watres.2020.116219.
45. Xu, B.; Zhou, J.L.; Altaee, A.; Ahmed, M.B.; Johir, M.A.H.; Ren, J.; Li, X. Improved Photocatalysis of Perfluorooctanoic Acid in Water and Wastewater by Ga2O3/UV System Assisted by Peroxymonosulfate. *Chemosphere* **2020**, *239*, 124722, doi:10.1016/j.chemosphere.2019.124722.
46. Gu, P.; Zhang, C.; Sun, Z.; Zhang, H.; Zhou, Q.; Lin, S.; Rong, J.; Hoffmann, M.R. Enhanced Photoreductive Degradation of Perfluorooctanesulfonate by UV Irradiation in the Presence of Ethylenediaminetetraacetic Acid. *Chem. Eng. J.* **2020**, *379*, doi:10.1016/j.cej.2019.122338.
47. Azarpira, H.; Abtahi, M.; Sadani, M.; Rezaei, S.; Atafar, Z.; bay, A.; Mohseni, S.M.; Sarkhosh, M.; Shanbedi, M.; Alidadi, H.; et al. Photo-Catalytic Degradation of Trichlorophenol with UV/Sulfite/ZnO Process, Simultaneous Usage of Homogeneous Reductive and Heterogeneous Oxidative Agents Generator as a New Approach of Advanced Oxidation/Reduction Processes (AO/RPs). *J. Photochem. Photobiol. A Chem.* **2019**, *374*, 43–51, doi:10.1016/j.jphotochem.2019.01.010.
48. Xu, T.; Zhu, Y.; Duan, J.; Xia, Y.; Tong, T.; Zhang, L.; Zhao, D. Enhanced Photocatalytic Degradation of Perfluorooctanoic Acid Using Carbon-Modified Bismuth Phosphate Composite: Effectiveness, Material Synergy and Roles of Carbon. *Chem. Eng. J.* **2020**, *395*, doi:10.1016/j.cej.2020.124991.
49. Olatunde, O.C.; Kuvarega, A.T.; Onwudiwe, D.C. Photo Enhanced Degradation of Polyfluoroalkyl and Perfluoroalkyl Substances. *Heliyon* 2020, *6*.
50. Winchell, L.J.; Ross, J.J.; Wells, M.J.M.; Fonoll, X.; Norton, J.W.; Bell, K.Y. Per- and Polyfluoroalkyl Substances Thermal Destruction at Water Resource Recovery Facilities: A State of the Science Review. *Water Environ. Res.* **2021**, *93*, 826–843, doi:10.1002/wer.1483.
51. Maga, D.; Aryan, V.; Bruzzano, S. Environmental Assessment of Various End-of-Life Pathways for Treating Per- and Polyfluoroalkyl Substances in Spent Fire-Extinguishing Waters. *Environ. Toxicol. Chem.* **2021**, *40*, 947–957, doi:10.1002/etc.4803.
52. Stoiber, T.; Evans, S.; Naidenko, O. V. Disposal of Products and Materials Containing Per- and Polyfluoroalkyl Substances (PFAS): A Cyclical Problem. *Chemosphere* **2020**, *260*, 127659, doi:10.1016/j.chemosphere.2020.127659.
53. Ahmed, M.B.; Alam, M.M.; Zhou, J.L.; Xu, B.; Johir, M.A.H.; Karmakar, A.K.; Rahman, M.S.; Hossen, J.; Hasan, A.T.M.K.; Moni, M.A. Advanced Treatment Technologies Efficacies and Mechanism of Per- and Poly-Fluoroalkyl Substances Removal from Water. *Process Saf. Environ. Prot.* **2020**, *136*, 1–14, doi:10.1016/j.psep.2020.01.005.
54. Solo-Gabriele, H.M.; Jones, A.S.; Lindstrom, A.B.; Lang, J.R. Waste Type, Incineration, and Aeration Are Associated with per- and Polyfluoroalkyl Levels in Landfill Leachates. *Waste Manag.* **2020**, *107*, 191–200, doi:10.1016/j.wasman.2020.03.034.
55. Dixit, F.; Dutta, R.; Barbeau, B.; Berube, P.; Mohseni, M. PFAS Removal by Ion Exchange Resins: A Review. *Chemosphere* 2021, *272*.
56. Mahinroosta, R.; Senevirathna, L. A Review of the Emerging Treatment Technologies for PFAS Contaminated Soils. *J. Environ. Manage.* 2020, *255*.
57. Lu, D.; Sha, S.; Luo, J.; Huang, Z.; Zhang Jackie, X. Treatment Train Approaches for the Remediation of Per- and Polyfluoroalkyl Substances (PFAS): A Critical Review. *J. Hazard. Mater.* **2020**, *386*, 121963, doi:10.1016/j.jhazmat.2019.121963.
58. Kowald, C.; Brorman, E.; Shankar, S.; Klemashevich, C.; Staack, D.; Pillai, S.D. PFOA and PFOS Breakdown in Experimental Sand, Laboratory-Grade Water, Investigation-Derived Groundwater and Wastewater Effluent Samples at 50 KGy Electron Beam Dose. *Radiat. Phys. Chem.* **2021**, *180*, 109323, doi:10.1016/j.radphyschem.2020.109323.
59. Feng, M.; Gao, R.; Staack, D.; Pillai, S.D.; Sharma, V.K. Degradation of Perfluoroheptanoic Acid in Water by Electron Beam Irradiation. *Environ. Chem. Lett.* **2021**, *19*, 2689–2694, doi:10.1007/s10311-021-01195-x.
60. Londhe, K.; Lee, C.-S.; Zhang, Y.; Grdanovska, S.; Kroc, T.; Cooper, C.A.; Venkatesan, A.K. Energy Evaluation of Electron Beam Treatment of Perfluoroalkyl Substances in Water: A Critical Review. *ACS ES&T Eng.* **2021**, *1*, 827–841, doi:10.1021/acsestengg.0c00222.
